# Jasmonate promotes auxin-induced adventitious rooting in dark-grown *Arabidopsis thaliana* seedlings and stem thin cell layers by a cross-talk with ethylene signalling and a modulation of xylogenesis

**DOI:** 10.1186/s12870-018-1392-4

**Published:** 2018-09-06

**Authors:** Laura Fattorini, Bettina Hause, Laurent Gutierrez, Angela Veloccia, Federica Della Rovere, Diego Piacentini, Giuseppina Falasca, Maria Maddalena Altamura

**Affiliations:** 1 Dipartimento di Biologia Ambientale, Sapienza Università di Roma, P.le Aldo Moro 5, I-00185 Rome, Italy; 2 Leibniz Institute of Plant Biochemistry, Department of Cell and Metabolic Biology, Weinberg 3, D06120, Halle (Saale), Germany; 3 CRRBM, SFR Condorcet FR CNRS 3417, Université de Picardie Jules Verne, Amiens, France

**Keywords:** Adventitious rooting, ARF17, EIN3/EIL1, Ethylene, Jasmonate, Thin cell layers, Xylogenesis

## Abstract

**Background:**

Adventitious roots (ARs) are often necessary for plant survival, and essential for successful micropropagation. In *Arabidopsis thaliana* dark-grown seedlings AR-formation occurs from the hypocotyl and is enhanced by application of indole-3-butyric acid (IBA) combined with kinetin (Kin). The same IBA + Kin-treatment induces AR-formation in thin cell layers (TCLs). Auxin is the main inducer of AR-formation and xylogenesis in numerous species and experimental systems. Xylogenesis is competitive to AR-formation in Arabidopsis hypocotyls and TCLs. Jasmonates (JAs) negatively affect AR-formation in de-etiolated Arabidopsis seedlings, but positively affect both AR-formation and xylogenesis in tobacco dark-grown IBA + Kin TCLs. In Arabidopsis the interplay between JAs and auxin in AR-formation vs xylogenesis needs investigation. In de-etiolated Arabidopsis seedlings, the Auxin Response Factors ARF6 and ARF8 positively regulate AR-formation and ARF17 negatively affects the process, but their role in xylogenesis is unknown. The cross-talk between auxin and ethylene (ET) is also important for AR-formation and xylogenesis, occurring through EIN3/EIL1 signalling pathway. EIN3/EIL1 is the direct link for JA and ET-signalling. The research investigated JA role on AR-formation and xylogenesis in Arabidopsis dark-grown seedlings and TCLs, and the relationship with ET and auxin. The JA-donor methyl-jasmonate (MeJA), and/or the ET precursor 1-aminocyclopropane-1-carboxylic acid were applied, and the response of mutants in JA-synthesis and -signalling, and ET-signalling investigated. Endogenous levels of auxin, JA and JA-related compounds, and *ARF6*, *ARF8* and *ARF17* expression were monitored.

**Results:**

MeJA, at 0.01 μM, enhances AR-formation, when combined with IBA + Kin, and the response of the early-JA-biosynthesis mutant *dde2–2* and the JA-signalling mutant *coi1–16* confirmed this result*.* JA levels early change during TCL-culture, and JA/JA-Ile is immunolocalized in AR-tips and xylogenic cells. The high AR-response of the late JA-biosynthesis mutant *opr3* suggests a positive action also of 12-oxophytodienoic acid on AR-formation*.* The crosstalk between JA and ET-signalling by EIN3/EIL1 is critical for AR-formation, and involves a competitive modulation of xylogenesis. Xylogenesis is enhanced by a MeJA concentration repressing AR-formation, and is positively related to *ARF17* expression.

**Conclusions:**

The JA concentration-dependent role on AR-formation and xylogenesis, and the interaction with ET opens the way to applications in the micropropagation of recalcitrant species.

**Electronic supplementary material:**

The online version of this article (10.1186/s12870-018-1392-4) contains supplementary material, which is available to authorized users.

## Background

Adventitious roots (ARs) are post-embryonic roots formed by various aerial organs. In numerous species, ARs contribute to plant anchorage and uptake of water and mineral nutrients from the soil. AR-formation is also a key step in the vegetative propagation of agronomically important crop species [1]. In the cuttings, excision rapidly alters the levels of auxin, the main AR-inducer, and leads to the production of other phytohormones, such as jasmonates (JAs) and ethylene (ET) [2]. In Arabidopsis seedlings, continuous darkness favors hypocotyl elongation and AR-formation [3], and the ARs are initiated by anticlinal divisions in the hypocotyl pericycle [4]. However, the founding tissue of ARs differs depending on the system used [5]. The stem endodermis is the founding tissue of ARs in Arabidopsis dark-grown thin cell layer (TCL) explants cultured with indole-3-butyric acid (IBA, at 10 μM) with/without cytokinin [kinetin (Kin) at 0.1 μM] [6, 7]. The TCL is formed by the tissues external to the vascular system of the inflorescence stem, and does not contain any auxin at culture onset [7]. Interestingly, AR-formation from intact dark-grown hypocotyls and TCLs of Arabidopsis is enhanced by the same IBA + Kin treatment [8], and is characterized by the same gene expression and auxin accumulation phases [8, 9]. IBA is a natural precursor of indole-3-acetic acid (IAA), and both in intact hypocotyls and TCLs must be converted into IAA to promote AR-formation [7, 10].

The ectopic formation of xylary cells (xylogenesis) may occur *in planta* and in in vitro cultured explants [11, 12], and in both cases is enhanced by exogenous auxins, alone or combined with cytokinin [9, 13, 14]. Xylogenesis is a competitive program to AR-formation in numerous species and cuttings [14, and references therein], including Arabidopsis dark-grown hypocotyls and TCLs [9, 11].

Jasmonates affect a lot of morphogenic processes [15]. When applied exogenously, methyl jasmonate (MeJA) is preferred to JA because of its easier cell membrane crossing ability and its rapid de-methylation to produce free JA [16]. The effects of JA on AR-formation are contradictory. For example, in petunia cuttings reduced levels of JA and its bioactive conjugate (+)-7-*iso*-jasmonoyl-L-isoleucine (JA-Ile) result into reduced AR-formation [17]. Moreover, in tobacco and Arabidopsis dark-grown IBA +/− Kin-cultured TCLs, AR-formation is enhanced by submicromolar concentrations of MeJA (0.1 μM and 0.01 μM, respectively), with increases in endogenous JA levels preceding the AR-process [7, 16]. However, a negative role of JA on AR-formation has been reported in de-etiolated intact hypocotyls of Arabidopsis [18].

Information about JA effect on xylogenesis is limited. In Arabidopsis seedlings grown under a light/dark regime, JA induces ectopic xylem formation in the primary root, but this promotion is antagonized by cytokinin [19]. In dark-grown tobacco TCLs, JA coming from MeJA application enhances xylogenesis, but this occurs in the presence of IBA + Kin [16].

The Arabidopsis mutants *delayed-dehiscence2–2* (*dde2–2)* [20] and *oxophytodienoate- reductase3* (*opr3*) [21] are defective in JA-biosynthesis. In *dde2–2* a transposon insertion disrupts the locus encoding allene oxide synthase, causing an early stop in JA-synthesis. In *opr3*, a T-DNA insertion disrupts the *OPR3* locus and the mutant is defective in OPR3, which converts 12-oxophytodienoic acid (OPDA) into the first precursor of JA. This mutant exhibits minor amounts of JA, but high amounts of OPDA [22], which might cause JA-independent responses [15]. The *coronatine insensitive1–16* (*coi1–16*) mutant is JA-insensitive [23]. The JASMONATE ZIM-DOMAIN (JAZ) proteins are the target of COI1 [24], supporting that COI1–JAZ is a co-receptor for JA-perception.

In de-etiolated Arabidopsis seedlings, the Auxin Response Factors (ARFs) ARF6 and ARF8 mediate auxin signalling at transcriptional level [25], and function as positive AR-regulators, whereas ARF17 as negative AR-regulator [26]. ARF6 and ARF8 promote JA production [27], and light positively regulates *ARF6/ARF8* expression and negatively *ARF17* expression [26], whereas continuous darkness effect on *ARFs* is unknown.

Auxin-ET crosstalk has been shown to be important in primary and lateral root development [28, 29], with interdependency of the two phytohormones at synthesis, transport and signalling levels. In Arabidopsis dark-grown seedlings, 1-aminocyclopropane-1-carboxylic acid (ACC), the precursor of ET, reduces AR-formation, when applied alone at 0.1 μM, but when applied with IBA (10 μM) enhances it. Because ACC enhances the endogenous levels of IAA and reduces those of IBA, a promoting action of ET on AR-formation by favouring the conversion of exogenous IBA into IAA has been proposed *in planta* [10].

ETHYLENE INSENSITIVE 3 (EIN3), and its homologous EIN3-LIKE 1 (EIL1), are the central transcription factors controlling the majority of ET responses [30]. Both ACC and ET activate EIN3/EIL1 by promoting their protein accumulation [31]. In dark-grown Arabidopsis seedlings, the response of *ein3eil1* double mutant demonstrates that ET is involved in both IBA-induced AR-formation and xylogenesis by the EIN3/EIL1 signalling pathway, however how IBA interacts with this network needs further investigation [10, 11]. EIN3/EIL1 are also the direct link for JA and ET-signalling, physically interacting with at least three JAZ members [32]. In accordance, *ein3eil1* is insensitive to both JA and ET in the induction of pathogen-responsive gene expression and root hair development [31].

All together, the research was aimed to investigate JA role on AR-formation and xylogenesis in dark-grown Arabidopsis seedlings and TCLs, and the relationship with ET and auxin. To that end, the responses to MeJA of intact seedlings and TCLs, and of mutants in JA synthesis and signalling were investigated, also recurring of hormonal quantifications and histological analyses. To shed light on the possible interaction between JA and ET, exogenous ACC was applied with/without MeJA to the JA-mutants and to the ET-mutant *ein3eil1*. To evaluate a possible involvement of auxin signalling by the ARF network in AR-formation vs xylogenesis, the expression of *ARF6*, *ARF8*, and *ARF17* was also examined in the presence/absence of MeJA.

Results uncover a critical function of the crosstalk between JA and ET signalling by EIN3/EIL1 in the control of AR-formation, involving a competitive modulation of xylogenesis. The expression of *ARF17* is positively associated with xylogenesis, and is enhanced by the MeJA concentration (10 μM) which represses AR-formation.

## Methods

### Material and growth conditions of seedlings and adult plants

Seeds of Arabidopsis *dde2–2* [20] (provided by Beat Keller, University of Zurich, Switzerland), of *opr3* [21] (provided by John Browse, Washington State University, USA), and of *coi1–16* [23] (provided by John G. Turner, University of East Anglia, UK), mutants and of the corresponding wild types (WTs) (Col, Ws, Col-gl1, respectively) were sterilized in 10% commercial hypochlorite - based bleach (5.6% active chlorine) for 10 min, washed three times in sterile distilled water and sown in square Petri dishes (12 × 12 cm; 15 seeds per dish) on full-strength MS [33] medium, containing 1% (*w*/*v*) sucrose (Sigma-Aldrich), 0.55 mM myo-inositol (Fluka), 0.1 μM thiamine-HCl (pH 5.7) (HF control medium), and 0.8% agar (Sigma-Aldrich), in the absence or presence of MeJA at concentrations of 0.01 μM, 0.1 μM, and 10 μM. Alternatively, seeds of the same lines were sown on the MS “rooting medium” used for thin cell layers (TCLs) culture (see below), containing 10 μM IBA (Merck) and 0.1 μM Kin (Sigma-Aldrich) (IBA + Kin medium), adding or not MeJA at the same concentrations as above. Seeds of *ein3eil1* mutant [31] (provided by Hongwei Guo, Peking University, China), and of its WT Col-0 were sterilized as above, and sown in dishes containing IBA + Kin medium, adding or not ACC at 0.1 μM. The HF media were sterilized by autoclaving at 120 °C for 20 min. IBA and Kin were added to the media taking the appropriate volume from stock-solutions (10^− 3^ M and 10^− 2^ M, respectively) before autoclaving. Sterile stock-solutions of MeJA 10^− 3^ M and of ACC 10^− 3^ M were prepared by filtering (with a 0.22 μm pore filter) and the appropriate volume was taken to reach the final concentration in the already autoclaved medium. Before adding MeJA or ACC, the temperature of the media was allowed to decrease to about 45–50 °C.

After a stratification lasting three days, at 4 °C under darkness [34], and exposure to white light for 6 h to induce germination (germination procedure, according to [3]), the dishes were placed in vertical position, in order to maintain the seedlings constantly in contact with the agar medium [3], and exposed to continuous darkness for 22 days after stratification (Days After Stratification, DAS), at 22 ± 2 °C. Seedlings were fixed in 70% ethanol until observation under light microscopy (LM).

*ARF6::GUS*, *ARF8::GUS,* and *ARF17::GUS* transgenic seedlings from seeds [26] (provided by Catherine Bellini, University of Umeå, Sweden) were grown in vitro for 22 DAS on HF control medium, adding or not 0.01 μM MeJA, under darkness. The sowing and the growth conditions were the same as above.

The plants used as the source of TCLs, belonging to *dde2–2, opr3, coi1–16, ein3eil1* mutants and their WTs, and to *ARF6::GUS*, *ARF8::GUS*, and *ARF17::GUS* transgenic lines, were grown in soil in a growth chamber, under long days, at 22 ± 2 °C, 70% humidity and white light (22 W/m^2^ intensity) for 40 days after seed germination [7].

### TCL culture

TCL (about 0.5 × 8 mm) explants, composed by epidermis, three layers of cortical parenchyma, endodermis and 1–2 layers of fibers [8], were excised aseptically from the inflorescence stem internodes of 30 randomly chosen plants per genotype (*dde2–2*, *opr3*, *coi1–16*, *ein3eil1* mutants and their WTs) and placed, epidermal side up, in Magenta type jars (10 TCLs per jar) containing full-strength MS medium supplemented with 0.55 mM myo-inositol (Fluka), 0.1 μM thiamine-HCl (Sigma-Aldrich), 1% sucrose, 0.8% (*w*/*v*) agar, 10 μM IBA plus 0.1 μM Kin (rooting medium; [6]). The pH was adjusted to 5.7 with 1 M NaOH before autoclaving. For *dde2–2*, *opr3*, *coi1–16* (and their WTs) TCL culture, MeJA was added to IBA + Kin medium at 0.01 μM, 0.1 μM, or 10 μM, while, for *ein3eil1* (and its WT) TCL culture, it was added at 0.01 μM only. ACC at 0.1 μM, alone or combined with 0.01 μM MeJA, was added to the IBA + Kin medium in TCL culture of *dde2–2*, *coi1–16*, *ein3eil1* mutants and their WTs. The IBA + Kin medium (without MeJA, and/or ACC) was always used as a control. A medium without IBA and Kin, but containing MeJA at all the concentrations described above, was preliminarily used to test the response of the TCLs of all the genotypes. The explants were cultured for 15 days at 22 ± 2 °C under continuous darkness. At the end of the culture, 60 explants per genotype and treatment were observed under the stereomicroscope for root scoring. Three complete sets of experiments, each using different sets of plants cultured under the same conditions, were carried out with very similar results.

50–100 mg of Ws and *opr3* TCLs per replicate, cultured with or without 0.01 μM MeJA, were harvested at days 0, 1, 3 and 5 for hormone quantification, and 20 mg of TCLs of the same genotypes, cultured with or without 0.01, 0.1 or 10 μM MeJA, were harvested at day 15 for RT-qPCR analyses, as described below. Finally, 10 Ws and *opr3* TCLs, cultured with 0.01 μM or 10 μM MeJA or without the compound, were harvested at days 8 and 15 and used for the JA/JA-Ile immunolocalization procedure (see below). Other samples were used for histological analyses (see below).

Thirty TCLs per *ARF6::GUS, ARF8::GUS*, and *ARF17::GUS* randomly coming from each of three experimental sets of transgenic plants were cultured in vitro on the IBA + Kin medium, supplemented or not with 0.01 μM, 0.1 μM or 10 μM MeJA.

### Root scoring and measurement of hypocotyl length

At 22 DAS, Arabidopsis seedlings were fixed in 70% ethanol, whole-mounted on slide glasses, and adventitious root primordia (ARPs) and ARs were counted along the hypocotyl by observations under LM (ZEISS Axiolab HBO 50). The images were acquired in digital form using a LEICA DFC 320 camera applied to the microscope by the LEICA IM1000 Image Manager software. Hypocotyl length was measured from images acquired in digital form using a LEICA MZ8 stereomicroscope equipped with a ZEISS AxioCam camera by the AxioVision Release 4.7.2 software.

Root productivity on TCLs was evaluated under the stereomicroscope after 15 days of culture, by examining 60 explants per genotype, treatment and experimental set of plants, and the images were acquired in digital form as for the hypocotyl. Data from one replicate are shown on the text.

### Histological analysis

Ten samples per set of experiments of Ws and *opr3* TCLs cultured with/without 0.01 μM and 10 μM MeJA, and of Col-0 TCLs cultured with/without 0.1 μM ACC, were harvested at days 3, 5, 8 (Ws and *opr3*), and at day 15 (Col-0, Ws and *opr3*), dehydrated by a graded ethanol series and embedded in Technovit 7100 (Heraeus Kulzer, Germany). The embedded samples were longitudinally sectioned at 6 μm with the MICROM HM 350 SV microtome and stained with toluidine blue 0.05% (*w*/*v*) for LM observations. The histological images of the TCLs were acquired in bright field using a LEICA DC500 camera applied to a Leica DMRB microscope by LEICA IM1000 Image Manager software.

### Histochemical analysis of GUS activity

The expression pattern of the reporter gene *uidA* under the control of *ARF6*, *ARF8* or *ARF17* promoters was examined in the transgenic *ARF6::GUS*, *ARF8::GUS* and *ARF17::GUS* TCLs, cultured for 15 days under darkness on the IBA + Kin medium in the presence/absence of 0.01 μM or 0.1 μM MeJA. The histochemical GUS assay was performed according to [8], with minor modifications. The samples were immersed in acetone 80% already cold (− 20 °C) and placed at − 20 °C for 20 min, then washed three times with distilled water, before the histochemical procedure. After incubation at 37 °C in the dark for 4 h, the GUS buffer was removed and replaced with ethanol 70%. Twenty samples for genotype, treatment, and set of experiments were then observed under the stereomicroscope. Moreover, 10 TCLs of each transgenic line were dehydrated and embebbed in resin as above, longitudinally sectioned at 12 μm intervals with the automatic microtome MICROM HM 350 SV, and observed under LM. The GUS assay was performed also on 20 seedlings per each transgenic line grown in vitro, in the absence/presence of 0.01 μM MeJA, under darkness for 22 DAS. The seedlings were fixed in ethanol 70% before their observation under LM. The images of the transgenic TCLs and whole-mount seedlings were acquired in digital form as previously described. The GUS expression pattern was very similar among the seedlings of each replicate, and also among seedlings from different replicates.

### Quantification of IAA, IBA and jasmonates

For IAA and IBA determinations, frozen plant material (50–100 mg) was homogenized with 10 ml methanol containing 50 ng of (^13^C_6_)IAA (Cambridge Isotope Laboratories) and (^13^C_8_,^15^N_1_)IBA (kindly provided by J. Cohen, University of Minnesota, USA) as internal standards. The homogenate was subjected to a column filled with 3 ml DEAE-Sephadex A25 (Amersham Pharmacia Biotech AB, Sweden). The column was washed with 3 ml methanol and with 3 ml 0.1 M acetic acid in methanol. Fractions with 3 ml of 1 M, 3 ml 1.5 M, and 3 ml 3 M acetic acid in methanol were combined, evaporated and separated by preparative HPLC (Eurospher 100-C18, 5 μm, 250 × 4 mm, Knauer, Germany) using solvent A (MeOH) and solvent B (0.2% acetic acid in H_2_O) in a gradient of 40% A to 100% A. Fraction at R_t_ 8 to 10 min was collected, evaporated, methylated with ethereal diazomethane and subjected to gas chromatography-mass spectrometry (GC-MS) as described by [35]. Quantification was done according to [36] using fragments *m/z* 136 (^13^C_6_)IAA-Me, *m/z* 130 (IAA-Me), *m/z* 139 (^13^C_8_,^15^N_1_)IBA-Me and *m/z* 130 1(IBA-Me).

Contents of OPDA, JA, and JA-Ile were quantified using a standardized ultraperformance liquid chromatography–tandem mass spectrometry (UPLC-MS/MS)-based method according to [37]. In brief, about 50 mg of frozen plant material was homogenized and extracted with 500 μl pure methanol supplied with [^2^H_5_]OPDA, [^2^H_6_]JA, and [^2^H_2_] JA-Ile (50 ng each) as internal standards. After centrifugation, the supernatant was diluted with 9 volumes of water and subjected to solid phase extraction on HR-XC (Chromabond, Macherey-Nagel) column. Elution was done with 900 μl acetonitrile. Ten μl of the eluate were subjected to UPLC-MS/MS according to [37]. The contents of OPDA, JA, and JA-Ile were calculated using the ratio of analyte and internal standard peak heights. Results were expressed as mean data and corresponding standard errors (SEs) from three biological replicates.

### Immunolocalization of JA/JA-Ile in TCLs

At days 8 and 15, Ws and *opr3* TCLs, cultured on IBA + Kin medium and treated or not-treated with 0.01 μM MeJA, were fixed, dehydrated and infiltrated as described by [38]. Briefly, the samples were fixed in 4% (*w*/*v*) 1-ethyl-3-(3-dimethyl aminopropyl)-carbodiimide hydrochloride (EDC, Merck) in PBS for 3 h at room temperature (RT) and, after dehydration in a graded series of ethanol, were infiltrated with polyethylene glycol 1500 (PEG 1500, Merck) at 55 °C. The PEG-embedded samples were hardened at RT and subsequently longitudinally sectioned (5 μm thick sections) with the Microm HM 350 SV microtome (Microm, Walldorf, Germany).

The immunolabeling procedure and the antibodies were according to [38]. The sections were incubated over-night with an anti-JA antibody (from rabbit), diluted in PBS containing 5% (*w*/*v*) BSA and 1% acetylated BSA, at 4 °C. After the treatment with the primary antibody, the sections were incubated with the secondary antibody, goat anti-rabbit-lgG coniugated with AlexaFluor488 (Invitrogen) diluted in 5% BSA/PBS, at 37 °C for 90 min. The green fluorescence is indicative for the presence of JA and JA-Ile in the cells, but not of MeJA or OPDA [38]. Positive controls were done by fixing Ws explants, cultured in the absence of MeJA, in a solution of 500 μM JA in 4% (w/v) EDC. Negative controls were done by omitting the primary antibody during the immunolocalization procedure (Additional file 1: Figure S1a-b).

The sections were observed with a Leica DMRB epifluorescence microscope equipped with the specific set of filters (EF 450–490 nm, DM 510 nm, SF 515 nm). The images were acquired with a Leica DC500 digital camera and analyzed with IM1000 image-analysis software (Leica).

### Quantitative RT-PCR (RT-qPCR) experiments

#### RNA isolation and cDNA synthesis

TCLs of both the *opr3* mutant and the corresponding WT (Ws) were cultured in vitro under the root-inductive hormonal conditions (10 μM IBA plus 0.1 μM Kin) and continuous darkness for 15 days, with or without MeJA at concentrations of 0.01, 0.1 or 10 μM. About 20 mg of TCLs per genotype and treatment were harvested at day 15, flash-frozen in liquid nitrogen, and ground into powder. Samples were prepared from three independent biological replicates. Total RNA was extracted using RNAqueous Kit (Ambion) according to the manufacturer’s instructions. After DNAse-treatment with TURBO DNA-free™ Kit (Ambion), reverse transcription was performed as follows: 2 μg of RNA were reverse-transcribed using 10^3^ U/ml of M-MuLV Reverse Transcriptase (Finnzymes) with 2.5 μg of random hexamers and 500 ng of oligo(dT)_18_ adapter primer in a total volume of 50 μl, and incubated for 60 min at 40 °C.The reaction was stopped by incubation at 70 °C for 15 min. After RNaseH (BioLabs) treatment, the reaction mixture was diluted by adding 700 μl of dH_2_O. All cDNA samples were tested by PCR using specific primers flanking an intron sequence to confirm the absence of genomic DNA contamination.

#### Quantitative RT-PCR experiment design

The transcript levels of *ARF6*/At1g30330, *ARF8*/At5g37020 and *ARF17*/At1g77850 (primer sequences listed in Additional file 2: Table S1) were assessed in three independent biological replicates by RT-qPCR, in assays with duplicate reaction mixtures (final volume, 20 μl) containing 5 μl of cDNA, 0.5 μM of both forward and reverse primers and 1× DyNAmo™ Flash SYBR® Green qPCR mix (Finnzymes). Quantitative RT-PCR experiments used a balanced randomized block design, as previously advised [39]. A LightCycler® (Roche) was used to acquire the CT values for each sample, i.e. the crossing threshold values, which are the number of PCR cycles required for the accumulated fluorescence signal to cross a threshold above the background [40]. The following standard protocol was applied for the amplification of each of the *ARF* cDNAs: an initial activation step of 10 min at 95 °C, followed by 40 cycles of 10 s at 95 °C, 15 s at 60 °C and 15 s at 72 °C. Each amplicon was first sequenced to ensure the specificity of the amplified sequence and, in order to check that the fluorescence signal was derived from the single intended amplicon in the succeeding runs, a melting curve analysis was added to each PCR program.

#### Quantitative RT-PCR data analysis

Relative standard curves describing the PCR efficiencies (E) for each primer pair were generated for each amplicon according to [41]. Normalization of quantitative RT-PCR was performed using reference genes (R), which were selected from [26] and validated in our experimental material according to [40]. *TIP41/*At4g34270 was the most stably expressed gene among the 11 tested, and thus was used to normalize the real-time RT-PCR data.

CT and E values were used to calculate expression using the formula E_T_^(CT^_A_^-CT^_B_^)^ / E_R_^(CT^_A_^-CT^_B_^)^, where (T) is the target gene and (R) the reference gene, (CT) is the crossing threshold value, (B) is related to cDNA from *opr3* mutant or Ws TCLs cultured with or without MeJA at different concentrations and (A) to cDNA from the calibrator [40]. The data are presented as relative to the MeJA-untreated Ws which is the calibrator and for which the gene expression ratio was set to 1. All the results are shown as mean data and corresponding SEs obtained from three biological replicates, each consisting of three technical replicates.

### Statistical analysis

Data were expressed as mean values (±SE). A normality test (Kolmogorov-Smirnov) was applied before the statistical analyses, using GraphPad Instat 3. Mean data were analyzed by Student’s *t* test (*P* < 0.05) to compare the effects of two different genotypes, or by one-way or two-way analysis of variance (ANOVA, *P* < 0.05) to compare the effects of different treatments and/or genotypes, or different treatments and days. If ANOVA showed significant effects, Tukey’s post-test was applied (GraphPad Prism 6.0). The significance of the differences between percentages was evaluated using χ^2^ test (*P* < 0.05). The three biological replicates of each experiment showed very similar results.

## Results

### Submicromolar MeJA concentrations reduce hypocotyl growth and enhance AR formation in IBA + Kin-grown WT seedlings and JA-mutants, except for coi1–16

The effect of exogenous MeJA was evaluated in mutants blocked in early (*dde2–2)* and late *(opr3)* steps of JA-biosynthesis, and in JA-signalling *(coi1–16)* grown in the presence of the environmental, hormonal, and culture conditions necessary for AR-formation in TCLs. As a premise, the effect of the same MeJA concentrations on seed germination was evaluated.

After stratification, the seeds were grown in vitro under continuous darkness with 10 μM IBA and 0.1 μM Kin, and either without MeJA (0 μM) or in the presence of 0.01 μM, 0.1 μM, and 10 μM MeJA. The presence of IBA and Kin never significantly reduced seed germination in comparison with the hormone-free (HF) treatment (Additional file 3: Figure S2a-b). The addition of either 0.01 μM MeJA or 0.1 μM MeJA, with/without IBA and Kin, did not affect significantly the germination in any genotype in comparison with the absence of the compound (Additional file 3: Figure S2a-b). By contrast, germination occurred at very low rates (0–17%) in all the genotypes in the presence of the highest concentration of MeJA (10 μM), combined or not with IBA and Kin, and when germination occurred, seedling growth was highly stunted, and AR-formation compromised, independently of the genotype.

At 22 DAS, all the IBA + Kin-grown seedlings of *dde2–2*, *opr3*, *coi1–16* and the corresponding WT genotypes, i.e., Col, Ws and Col-gl1, respectively, showed ARPs and ARs along the hypocotyl. For this reason, the mean hypocotyl length and the AR density were evaluated.

**Fig. 1:**
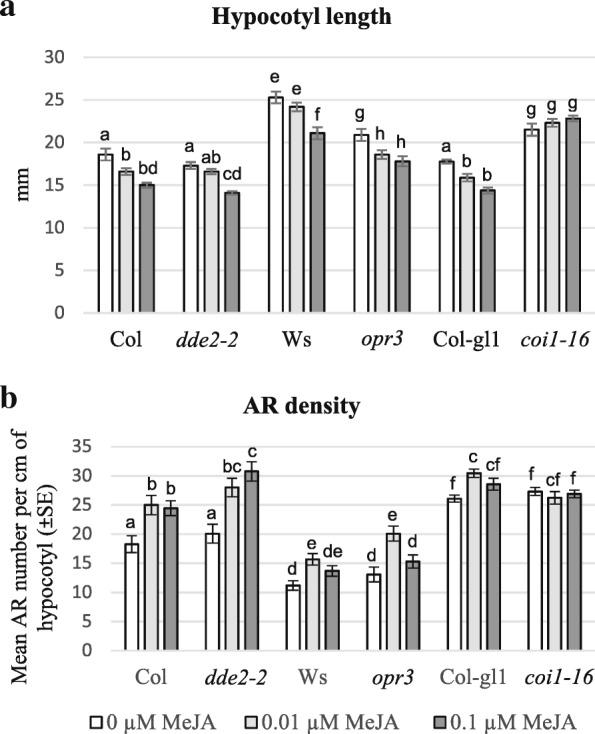
Hypocotyl length and AR-formation in dark-grown JA-mutant seedlings treated with IBA + Kin and MeJA. (**a**) Mean hypocotyl length (±SE) at 22 DAS of *dde2–2*, *opr3* and *coi1–16* mutants, and of their WTs (Col, Ws and Col-gl1, respectively), grown either in the absence of MeJA (0 μM MeJA) or with 0.01 μM or 0.1 μM MeJA. (**b**) Mean AR-density, i.e. mean ARP/AR number per cm of hypocotyl, (±SE) on the same seedlings as in (**a**). Significant differences between treatments within the same genotype, or between each mutant and its WT under the same treatment, at least at *P < 0.05* level*,* are indicated by different letters. The same letter shows no statistical difference. Further statistical details are described on the text. *N* = 30 (first replicate)

Except for *coi1–16* seedlings, which showed the same hypocotyl length with/without MeJA in accordance with the mutant insensitivity to MeJA/JA perception, in all other genotypes 0.1 μM MeJA caused a significant (*P* < 0.0001) reduction in hypocotyl length in comparison with its absence (0 MeJA, control), which however remained below 20% (Fig. 1a). A ten-fold lower concentration of MeJA (0.01 μM) showed a similar, but less pronounced, effect, that, within the same genotype and with respect to the control, was statistically significant only for two WT genotypes (Col and Col-gl1) (*P* < 0.05), and for *opr3* (*P* < 0.01) (Fig. 1a).

The IBA + Kin-treated WT seedlings produced a mean number of ARPs/ARs per cm of hypocotyl (AR density) significantly higher, by 8-fold in Col, 4-fold in Ws, and 11-fold in Col-gl, respectively, than the HF-cultured seedlings (Fig. 1b and Additional file 3: Figure S2c), confirming previous results for Col and Ws [8]. As shown in Fig. 1b, except for *coi1–16,* in which MeJA did not cause any change in AR-formation, the two submicromolar MeJA concentrations were effective in enhancing AR-response, with 0.01 μM MeJA inducing significant increases in all the other genotypes (*P <* 0.05 for Ws and Col-gl1, *P* < 0.001 for Col and *opr3*, *P* < 0.0001 for *dde2–2*) in comparison with the control treatment.

### 0.01 μM MeJA enhances AR-formation in TCLs of both WT and JA-mutants, except for coi1–16

To a deep insight into the effect of JA in AR-formation, the AR-response of TCLs excised from the stems of plants of all the genotypes was investigated under the same conditions as used for seedlings. MeJA was applied together with IBA + Kin at 0.01 μM and 0.1 μM, but also at 10 μM, because the latter concentration had caused some callus proliferation in tobacco TCLs cultured under the same conditions [16]. Arabidopsis WT TCLs (Col-0 and Ws genotypes) are known to be unable to produce ARs under HF conditions [6]. However, to verify whether MeJA was able per se to induce AR-formation, preliminarily the TCLs were cultured without hormones but with each MeJA concentration. Neither AR-formation nor callus-formation occurred independently of the genotype.

**Fig. 2:**
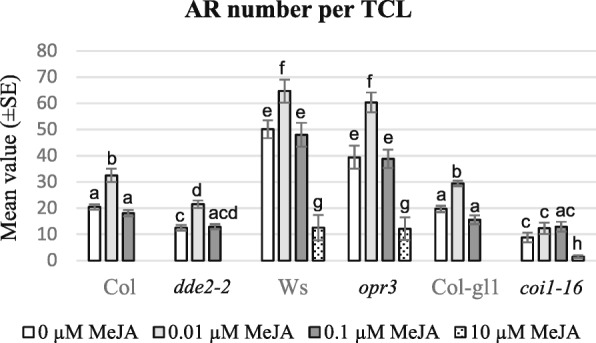
AR-formation in TCLs of JA-mutants cultured with IBA + Kin and MeJA under continuous darkness. Mean ARP/AR number (±SE) per TCL excised from *dde2–2*, *opr3*, *coi1–16* plants, and from their WTs (Col, Ws and Col-gl1, respectively), after 15 days of culture either in the absence (0 μM MeJA) or in the presence of 0.01 μM, 0.1 μM, and 10 μM MeJA. Significant differences between treatments within the same genotype, or between each mutant and its WT under the same treatment, at least at *P* < 0.05 level, are indicated by different letters. The same letter shows no statistical difference. Further statistical details are described in the text. *N* = 60 (first replicate)

**Fig. 3:**
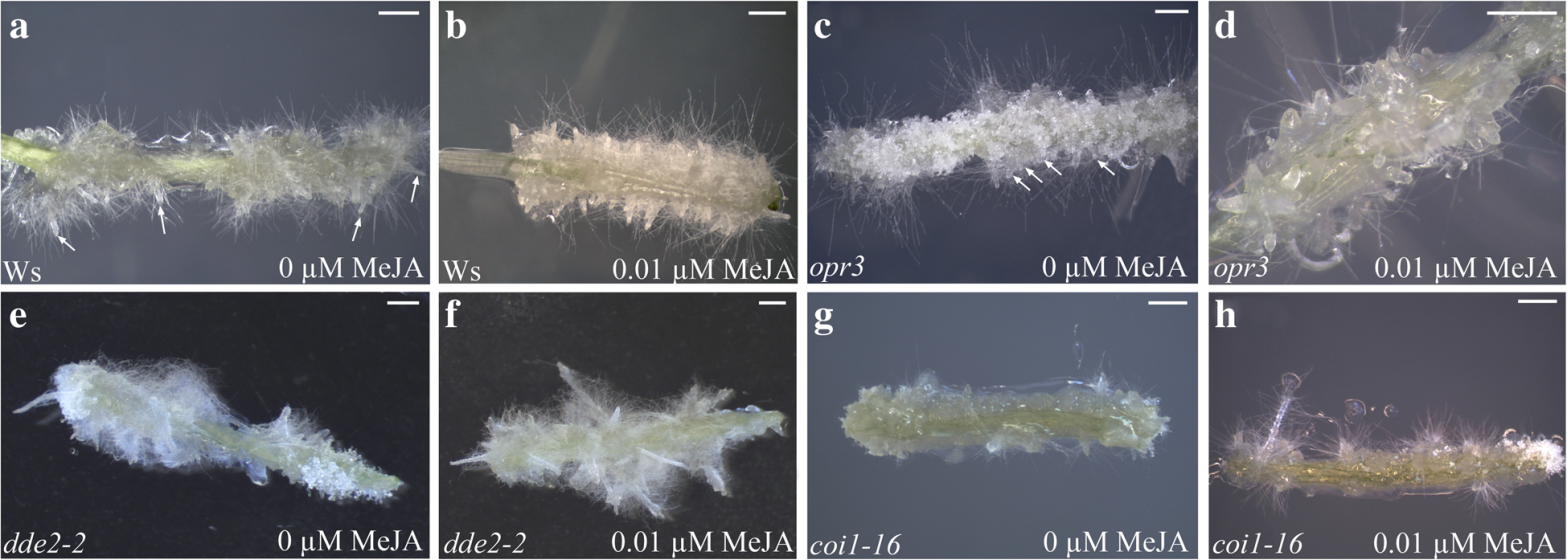
Macroscopic AR-response with/without 0.01 μM MeJA in IBA + Kin-cultured TCLs from JA-mutants. (**a-h**) Images under the stereomicroscope of the WT Ws (**a**-**b**), *opr3* (**c**-**d**), *dde2–2* (**e**-**f**), *coi1–16* (**g**-**h**) TCLs at the end of the in vitro culture (day 15) in the absence (**a**, **c**, **e**, **g**) or in the presence of 0.01 μM MeJA (**b**, **d**, **f**, **h**). (**a**) TCL with elongated ARs (arrows) and callus. (**b**) TCL with a production of ARs higher than in (**a**). (**c**) TCL with callus with ARPs (arrows) and elongating ARs. (**d**) Detail of a TCL portion showing numerous ARPs. (**e**) TCL producing fewer ARs than in (**f**). (**g-h**) Similar ARP/AR production in TCLs cultured either without (**g**) or with (**h**) MeJA. (Images from the first replicate). Bars = 1 mm

After 15 days of culture under IBA + Kin, the percentage of TCLs with ARPs/ARs and the mean number of macroscopic ARPs/ARs per explant was evaluated. In the absence of MeJA, the percentage of rooting explants was around 70% in Ws, *opr*3, Col, *dde2–2,* and Col-gl1 genotypes*,* and 40% in *coi1–16*. The presence of the two MeJA submicromolar concentrations did not change significantly these values. By contrast, 10 μM MeJA reduced the percentage of AR-forming TCLs by about 6 fold in Ws, *opr3*, and *coi1–16*, and nullified the AR-response in Col, *dde2–2* and Col-gl1 TCLs. The explants with no AR either remained unchanged in comparison with the culture onset or exhibited a little amount of callus. In the absence of MeJA there was some difference in the AR-mean number among the WT genotypes, and AR-formation was significantly higher in Ws than in Col and Col-gl1 (Fig. 2). The AR-number was significantly enhanced by the presence of 0.01 μM MeJA in comparison with 0 MeJA in all genotypes (*P* < 0.05 for *dde2–2* and Col-gl1, *P* < 0.01 for Col, *P* < 0.001 for Ws, and *P* < 0.0001 for *opr3*), except for *coi1–16* (Figs. 2 and 3). Among the WT TCLs, the highest increase was observed in Ws (Figs. 2 and 3a-b). By contrast, no significant difference in AR-number occurred between 0.1 μM MeJA and 0 MeJA for all genotypes (Fig. 2), and a strong reduction in AR-number was observed in the few genotypes (Ws, *opr3*, *coi1–16*) able to form ARs under 10 μM MeJA (Fig. 2). In all genotypes the TCLs with ARs also showed macroscopic callus formation (Fig. 3).

Independently of the treatment, *opr3* TCLs showed an AR-response similar to their WT Ws (Fig. 2), even if, differently from the WT, the majority of the roots remained at the ARP-stage (Fig. 3c-d). Differently from *opr3*, *dde2–2* TCLs showed an AR-production (Fig.3e-f) significantly lower than in their WT, i.e., Ws and Col, respectively, even when treated with 0.01 μM MeJA (*P* < 0.05 for both 0 and 0.01 μM MeJA, and Fig. 2). Having in mind that OPDA is present in *opr3* mutant and not in *dde2–2* mutant, the result suggests a positive interaction between JA deriving from exogenous MeJA and the endogenous OPDA in enhancing AR-formation. By contrast, the effects of JA and OPDA on AR elongation need further investigation.

Differently from the Col-gl1 TCLs, *coi1–16* TCLs did not show significant changes in AR production when treated with the submicromolar MeJA concentrations, and AR response was lower than the WT (*P* < 0.05 and *P* < 0.0001 under 0 and 0.01 μM MeJA, respectively, Figs. 2 and 3g-h).

**Fig. 4:**
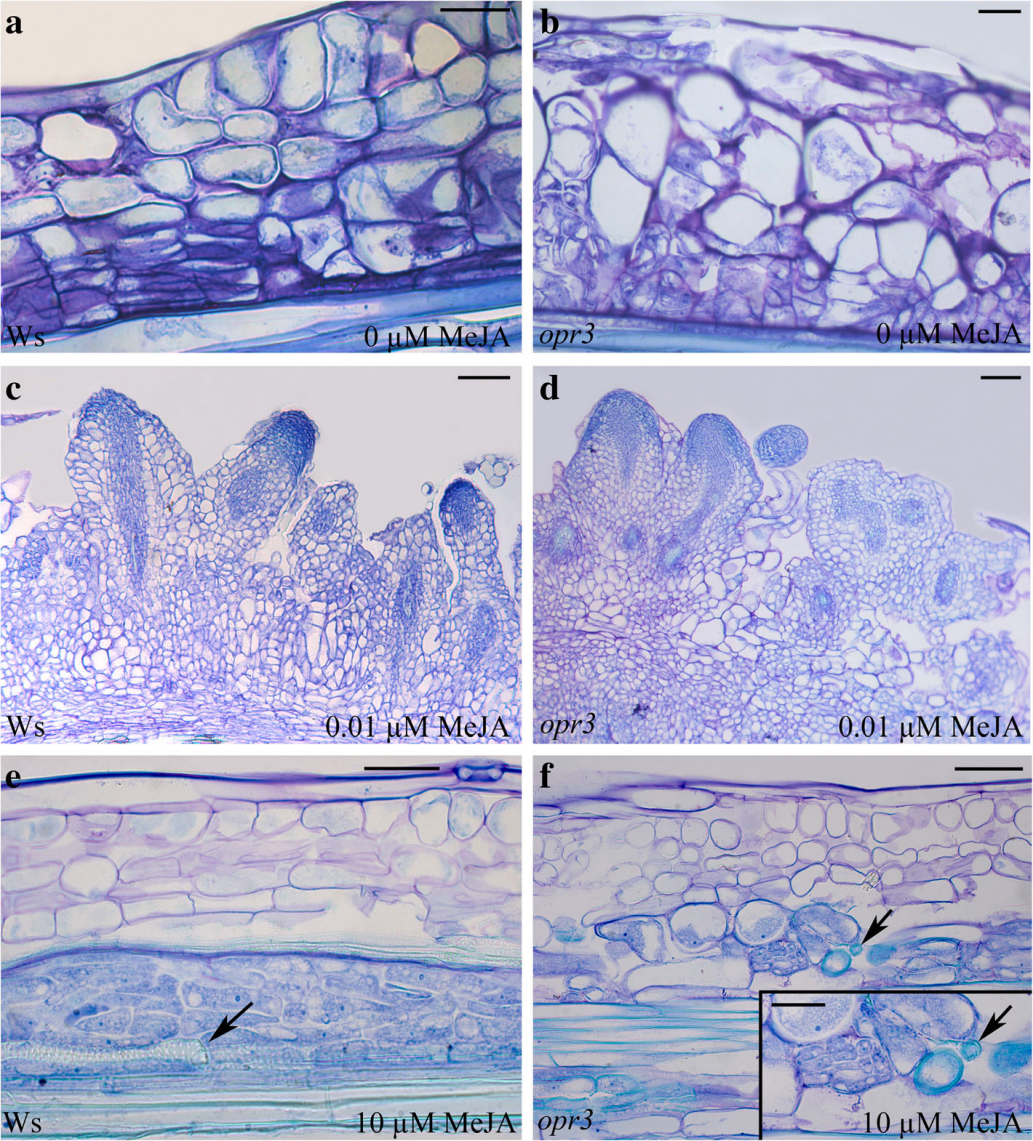
Histological images of Ws and *opr3* IBA + Kin*-*TCLs cultured with or without MeJA. (**a-f**) Longitudinal radial sections of Ws (**a, c**, **e**) and *opr3* (**b**, **d**, **f**) TCLs cultured in the absence of MeJA (**a**, **b**), or in the presence of either 0.01 μM MeJA (**c, d**) or 10 μM MeJA (**e**, **f**). (**a**) Cell divisions starting from the stem endodermis (day 3). (**b**) Meristematic cell clusters organized by the endodermis derivative cells (day 5). (**c**) Elongating ARs protruding from the explant (day 15). (**d**) ARPs (on the left, longitudinal section view) and ARs (on the right, transection view) at day 15. (**e**) Xylary elements (arrow) differentiating from the endodermis-derived cells (day 15). (**f**) Hypertrophic cells and de novo formed xylary elements (arrow) surrounding a meristematic cell cluster (magnified in the Inset) (day 15). Sections stained with toluidine blue. (Images from the first replicate). Bars = 50 μm (**a**, **b**, **e** and Inset in **f**), 100 μm (**c**, **d**, **f**)

Based on the similar trend, but the increased AR-production of Ws and *opr3* TCLs in comparison with all the other genotypes, and the presence of an AR-response even with 10 μM MeJA (Fig. 2), the TCLs of these genotypes were examined in detail through a histological analysis. With 0, 0.01 and 0.1 μM MeJA, the first cell divisions occurred in the endodermis at day 3 independently of the genotype (Fig. 4a), were followed by the organization of the first meristematic cell clusters at day 5 (Fig. 4b), and by the bulk of ARP/AR-formation between days 10 and 15 (Fig. 4c-d). In the same time interval, the endodermis derivatives of the TCLs treated with 10 μM MeJA either became hypertrophic or produced xylary elements (Fig. 4e-f) rather than ARPs.

### JA, OPDA and IAA levels change in WT and opr3 TCLs during the first days of culture

**Fig. 5:**
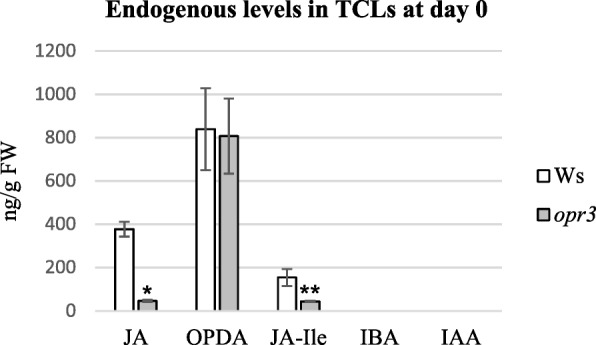
Endogenous levels of jasmonates and auxins in Ws and *opr3* TCLs soon after their excision. Mean values (±SE) of JA, OPDA, JA-Ile, IBA and IAA, expressed as ng/g FW, in Ws and *opr3* TCLs soon after their excision from the stem (day 0). *^,^ *P* < 0.001 difference with respect to the WT Ws; **^,^ *P* < 0.05 difference with respect to the WT Ws. No asterisk shows no significant difference in OPDA level between the two genotypes. N = 3 biological replicates

The similar results obtained with *opr3* and Ws TCLs under the macroscopic and histological analyses (Figs. 2 and 4) prompted us to investigate the endogenous levels of jasmonates and auxin in the same genotypes, and under the best treatment (0.01 μM MeJA) for AR-formation (Fig. 2). Neither IBA nor IAA were detected in Ws and *opr3* TCLs soon after the excision from the stem (i.e. day 0), in accordance with previous results [7]. By contrast, and possibly as a consequence of an excision-caused wounding reaction, JA, OPDA and JA-Ile were present (Fig. 5). The endogenous levels of JA and of JA-Ile were significantly higher in the WT than in the mutant (at *P* < 0.001 and *P* < 0.05, respectively), whereas OPDA levels were similar, with about 2-fold and 17-fold increases, respectively, in comparison with JA (Fig. 5).

**Fig. 6:**
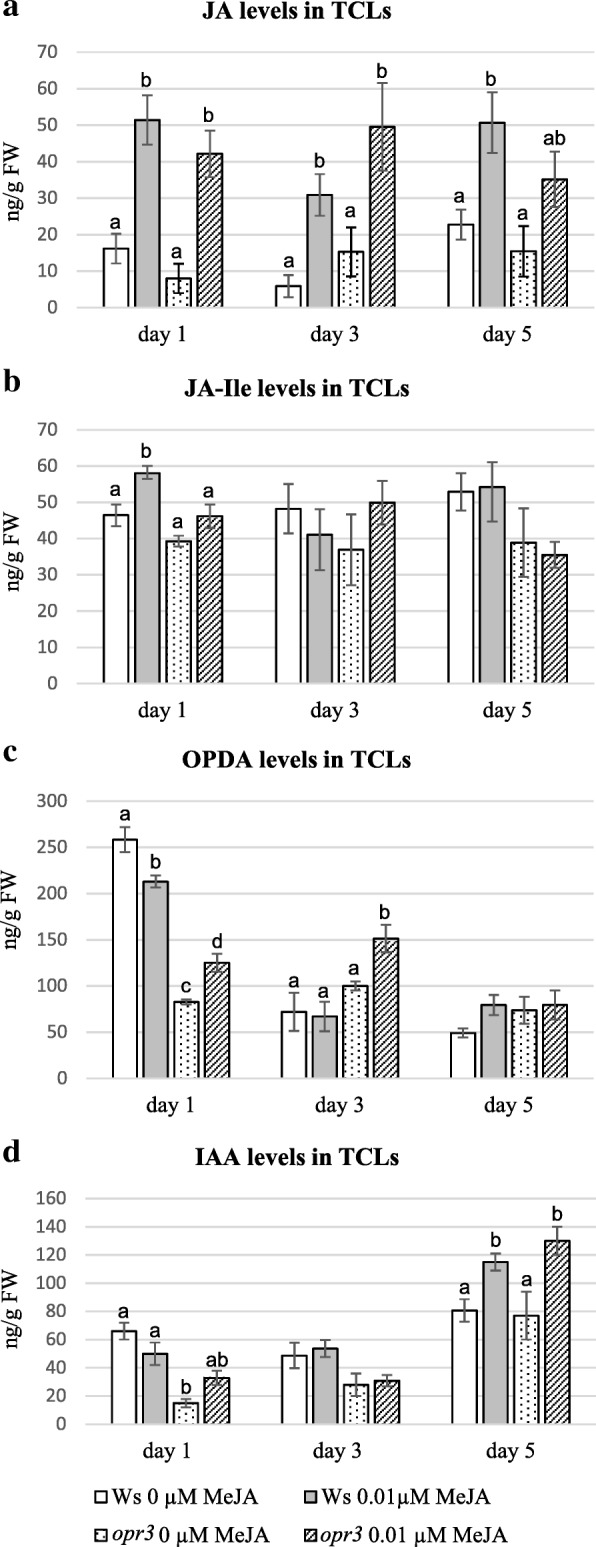
Endogenous levels of jasmonates and IAA during early-culture with/without MeJA in Ws and *opr3* IBA + Kin*-*cultured TCLs. (**a**-**d**) Mean values (±SE) of endogenous JA (**a**), JA-Ile (**b**), and OPDA (**c**), and IAA (**d**) expressed as ng/g FW, in Ws and *opr3* TCLs after 1, 3 and 5 days of culture under darkness with or without 0.01 μM MeJA. Significant differences at the same day between treatments within the same genotype, or between genotypes under the same treatment, at least at *P < 0.05* level*,* are indicated by different letters. No letter or the same letter indicates no statistical difference at the same day. Further statistical details are described in the text. N = 3 biological replicates

**Fig. 7:**
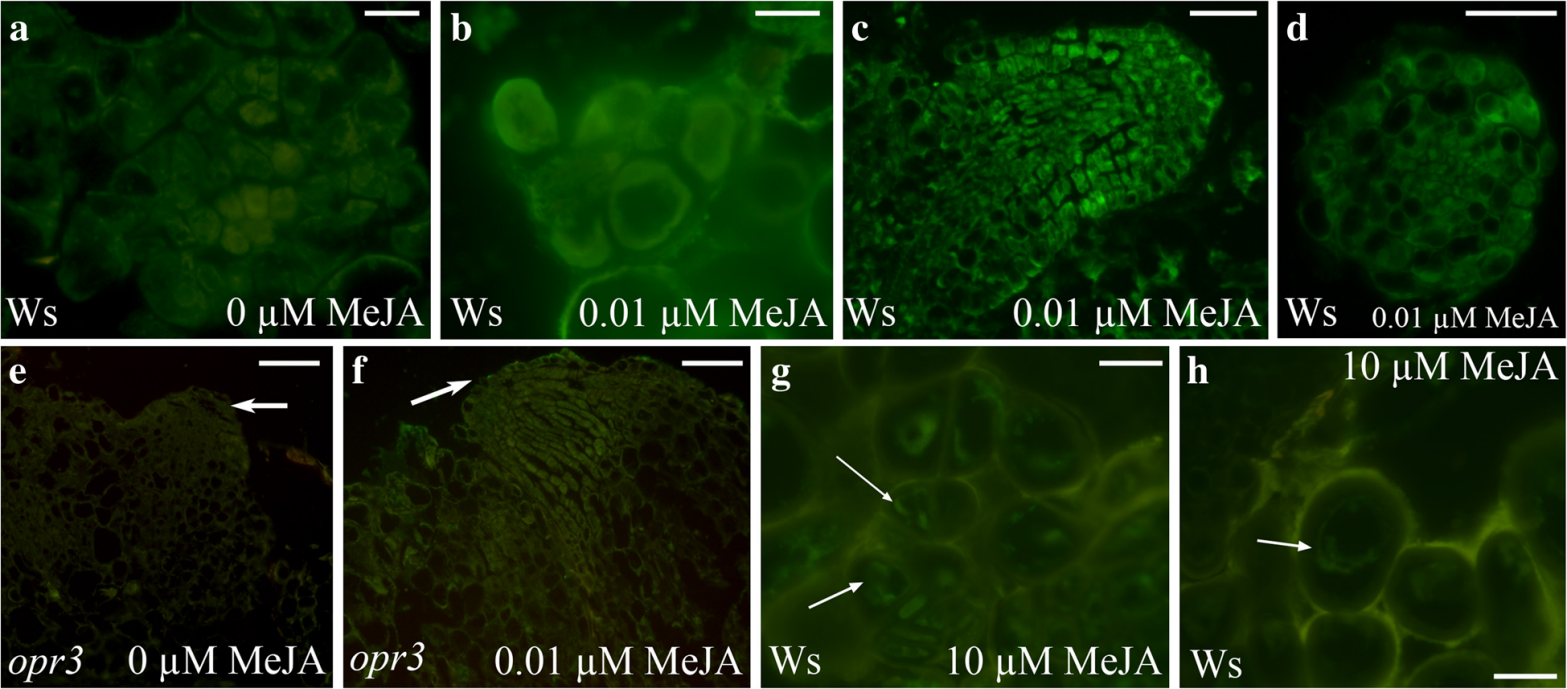
Immunolocalization of JA/JA-Ile in Ws and *opr3* IBA + Kin-cultured TCLs. (**a**-**h**) Immunocytochemical detection of JA/JA-Ile in Ws (**a**-**d**, **g**-**h**) and in the JA-deficient mutant *opr3* (**e**-**f**) longitudinal radial sections of TCLs, at days 8 and 15 of culture under darkness in the absence (**a**, **e**) or in the presence of 0.01 μM MeJA (**b**-**d**, **f**) or 10 μM MeJA (**g**-**h**). (**a**) Cells of a meristemoid showing the green epifluorescence signal of JA/JA-Ile immunodetection (day 8). (**b**) Cells of a meristematic clump showing an epifluorescence signal more intense than in (**a**) (day 8). (**c**) Detail of an AR tip (view in longitudinal section) showing apical cells with the signal (day 15). (**d**) Detail of an AR tip (view in transection) showing cells with the signal (day 15). (**e**) *opr3* ARP cells with no signal (arrow) in a TCL cultured without exogenous MeJA (day 15). (**f**) Faint signal in an *opr3* ARP (arrow) of a TCL cultured with 0.01 μM MeJA (day 15). (**g**) Weak signal (arrows) inside the cells of a differentiating xylary clump (day 15). (**h**) Weak signal (arrow) inside immature de novo formed xylary cells (day 15). (Images from the first replicate). Bars = 20 μm (**a**, **b**, **g**, **h**), 40 μm (**c**, **d**), 60 μm (**e**, **f**)

In the WT TCLs, the levels of JA, JA-Ile, and OPDA abruptly decreased at day 1 in culture in comparison with day 0 (Figs. 5 and 6), however, the treatment with 0.01 μM MeJA caused a JA (Fig. 6a) and JA-Ile (Fig. 6b) content significantly higher than without MeJA (*P* < 0.0001 for JA, *P* < 0.01 for JA-Ile). By contrast, OPDA levels were significantly (*P* < 0.001) lower with than without MeJA (Fig. 6c). In *opr3* MeJA-cultured TCLs, JA changed similarly to the WT both with and without MeJA (Fig. 6a), and JA-Ile showed a level similar to that of day 0 (Figs. 5 and 6b), without significant changes due to the presence of the compound (Fig. 6b).The level of OPDA in *opr3* TCLs was also significantly highly reduced in comparison with day 0 (Figs. 5 and 6c), but at a higher level without MeJA than with the compound (*P* < 0.01 difference, Fig. 6c). At day 3, i.e. when the first divisions occurred in the endodermis in both genotypes (Fig. 4a), JA levels became further reduced in the WT with/without MeJA, but remained significantly higher in the presence of MeJA than in its absence (*P* < 0.05 difference, Fig. 6a). In the WT, at the same day, the treatment with MeJA did not cause any significant change in JA-Ile and in OPDA levels in comparison with 0 MeJA (Fig. 6b-c), even if OPDA levels had become about 4-fold reduced in comparison with day 1 (Fig. 6c). At day 3, JA levels significantly (*P* < 0.01) increased in *opr3* TCLs treated with MeJA in comparison with the untreated ones, repeating the trend observed at day 1 (Fig. 6a). No change occurred in JA-Ile levels, as in the WT at the same day (Fig. 6b), but, differently from the WT, OPDA levels significantly (*P* < 0.05) increased with MeJA, in comparison with its absence, reaching a value significantly higher (*P* < 0.001) than in the WT under the same treatment (Fig. 6c). At day 5, i.e., the time of the first cell clusters formation (Fig. 4b), in the WT TCLs, JAlevel was again significantly higher (*P* < 0.01) in the presence of MeJA than in its absence, and similar to the level of day 1, whereas no significant increase occurred in *opr3* TCLs (Fig. 6a). The levels of JA-Ile and of OPDA were not significantly affected either by the genotype or the treatment (Fig. 6b-c). During the following days of culture, the presence of JA/JA-Ile in the WT and *opr3* explants, untreated or treated with 0.01 μM MeJA, was monitored by immunolocalization [38]. At day 8, in the absence of MeJA, JA/JA-Ile was detected in the cells of the meristematic cell clusters and meristemoids (Fig. 7a), but the signal was reinforced in the presence of 0.01 μM MeJA (Fig. 7b). The signal continued to be shown by all the cells in the ARP and AR tips (day 15) (Fig. 7c-d). A weak signal was instead detected in the ARPs of the *opr3* mutant TCLs, and mainly in the presence of 0.01 μM MeJA (Fig. 7e-f, arrows).

The immunolocalization of JA/JA-Ile in the WT TCLs cultured with 10 μM MeJA for 15 days showed signal presence in the xylary clumps (Fig. 7g) and in the single xylary cells (Fig. 7h) *de novo* formed at this concentration, in particular. The presence of a consistent JA level up to day 5 with 0.01 μM MeJA in both WT and *opr3* (Fig. 6a) suggested us to evaluate the IAA levels in the explants during the first five days of culture. As shown in Fig. 6d, in the WT and *opr3* TCLs cultured without MeJA, IAA was detected in the explants at day 1, but its level did not change significantly up to day 3, showing, instead, a significant increase (*P* < 0.05 for the WT and *P* < 0.01 for *opr3*) at day 5. The IAA trend was similar in the 0.01 μM MeJA-treated WT and *opr3* TCLs, with, however, a higher increase at day 5 (*P* < 0.001 for the WT, and *P* < 0.0001 for *opr3*) in comparison with the MeJA-untreated explants (Fig. 6d).At day 1, IBA was detected at higher amounts than IAA, both in WT (1970 ± 160 ng/g FW without MeJA and 1777 ± 390 ng/g FW with MeJA), and *opr3* explants (1313 ± 240 and 1266 ± 320 ng/g FW, respectively)**,** in accordance with the exogenous IBA supply**.** Up to day 5 the IBA trend remained similar to that of IAA. At day 5, Ws IBA values raised to 3284 ± 470 ng/g FW in the presence of 0.01 μM MeJA, and to 3052 ± 390 ng/g FW in the absence of MeJA, with both values not significantly different from the corresponding values in the mutant (not shown).

### ARF6 and ARF8 gene expression is not altered during AR-formation induced by MeJA, whereas ARF17 expression is enhanced by 10 μM MeJA and localizes in the xylogenic cells

It is known that in Arabidopsis ARF6 and ARF8 are essential for AR-formation in hypocotyls of light-grown seedlings, whereas ARF17 is an inhibitor of AR-formation under the same conditions [18, 26]. For these reasons, the activity of their promoters was monitored in the dark-grown seedlings and TCLs by the use of *ARF6::GUS*, *ARF8::GUS*, and *ARF17::GUS* transgenic lines.

**Fig. 8:**
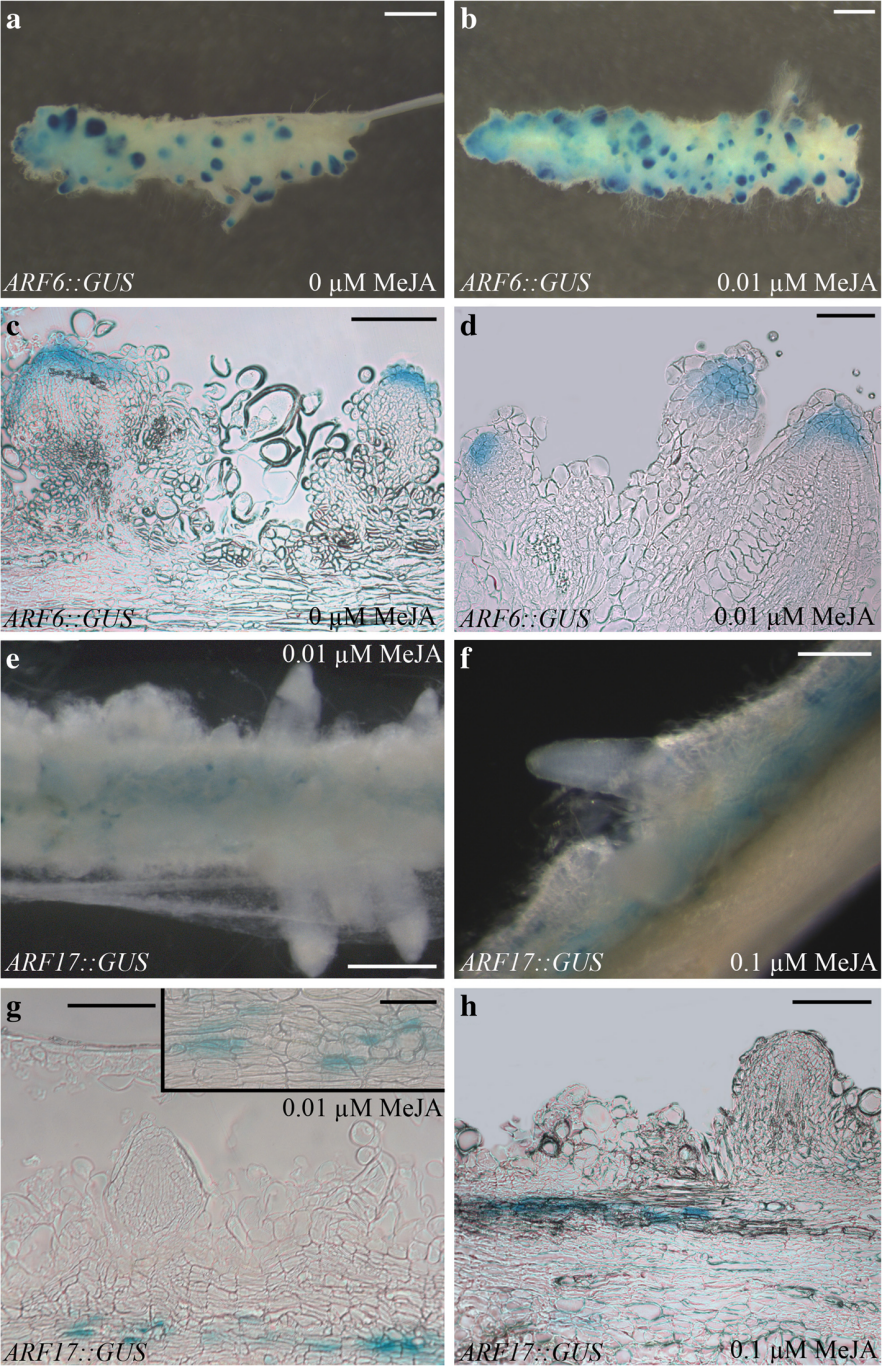
*ARF6::GUS* and *ARF17::GUS* expression patterns in IBA + Kin-TCLs cultured with or without MeJA. (**a**-**h**) Localization of the GUS signal in *ARF6::GUS* (**a**-**d**) and *ARF17::GUS* TCLs (**e**-**h**) cultured under darkness for 15 days in the absence of MeJA (0 μM MeJA) (**a**, **c**) and in the presence of 0.01 μM MeJA (**b**, **d**-**e**, **g**) or of 0.1 μM MeJA (**f**, **h**). (**a**) GUS signal in the ARPs and in the apex of elongated ARs. (**b**) TCL showing the same *ARF6::GUS* expression as in (**a**). The high presence of the signal is in accordance with the production of ARPs/ARs higher in (**b**) than in (**a**). (**c**-**d**) Localization of the GUS signal in the apical part of the ARPs. (**e-f**) *ARF17::GUS* expression absent in the ARs, but present along the explant. (**g**) *ARF17::GUS* expression localized in the cells differentiating into xylary elements, as magnified in the Inset. (**h**) *ARF17* GUS signal present in the strands of cells differentiating into xylary elements, but absent in the ARPs. (**a**-**b**, **e**-**f**) Stereomicroscope images, (**c**-**d**, **g**-**h**) Longitudinal radial sections of TCLs observed under LM. (Images from the first replicate). Bars = 50 μm (Inset in **g**), 100 μm (**d**), 200 μm (**c**, **f**-**h**), 500 μm (**e**), 1 mm (**a**, **b**)

Observations of the transgenic dark-grown seedlings at 22 DAS showed the presence of expression of *ARF6* and *ARF8*, but not of *ARF17*, in the ARP/AR tips (Additional file 4: Figure S3a-f), both with and without 0.01 μM MeJA. By contrast, *ARF17* was expressed in the vascular system of both the hypocotyls, lateral roots (LRs), and ARs, and again with and without MeJA (Additional file 4: Figure S3g-i). Observations of the TCLs cultured for 15 days under 0.01 and 0.1 μM MeJA, or in MeJA absence, showed the presence of the GUS signal in the ARP-tips of *ARF6::*GUS and *ARF8::*GUS TCLs, with a similar expression pattern, as exemplified for *ARF6* with/without 0.01 μM MeJA in Fig. 8a-d. The same as the TCLs of their background Columbia (Fig. 2), the explants of the transgenic lines were unable to grow and form ARs under 10 μM MeJA (data not shown), and for this reason, the GUS assay was not performed with these explants. The expression of *ARF17* was absent in the ARPs/ARs, but present in callused zones of the explants, independently of MeJA concentration (Fig. 8e-f). The histological analysis confirmed the macroscopic observations, and revealed that *ARF17* expression was localized in the xylogenic cells (Fig. 8g-h).

**Fig. 9:**
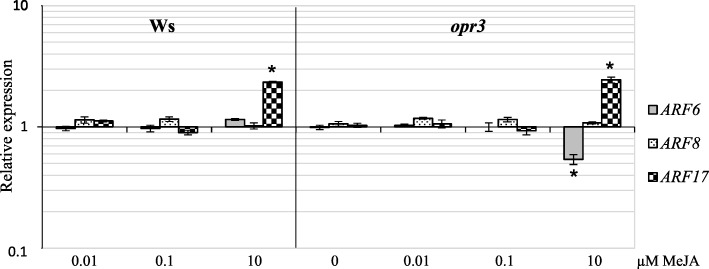
*ARF6*, *ARF8* and *ARF17* expression analysis in Ws and *opr3* TCLs by quantitative RT-qPCR. Quantification by RT-qPCR of *ARF6*, *ARF8*, *ARF17* transcript levels in Ws and *opr3* TCLs, cultured on IBA + Kin medium, without (0 μM MeJA) or with 0.01, 0.1 and 10 μM MeJA, under darkness for 15 days. Expression values of each gene are relative to the expression in the WT Ws TCLs cultured in absence of MeJA (calibrator), for which the value is set to 1. Transcript levels of each gene were normalized to the expression of *TIP41.* The asterisk indicates significant difference in gene expression (*P* < 0.01) of 10 μM MeJA compared to the same genotype without MeJA*.* Further statistical details are described on the text. Mean data and SEs were obtained from three biological replicates each consisting of three technical replicates

Because it is known that the stability of the transcripts of the three genes is under the control of specific miRNAs, at least under light [26, 42], their expression was also monitored by quantitative RT-PCR (RT-qPCR). The transcript levels were analyzed in the Ws TCLs and in those of the *opr3* mutant under all the MeJA concentrations, because of the high capability to form ARs of these TCLs, and the presence of an AR-response even with 10 μM MeJA (Fig. 2). Calibrating to 1 the expression of each gene in Ws explants cultured without MeJA (control TCLs), the RT-qPCR-analysis showed that the transcript levels of *ARF8* did not change significantly under MeJA treatments in comparison with control TCLs (Fig. 9). The same occurred for *ARF6* expression except in *opr3* TCLs treated with the highest MeJA concentration, where the transcript level decreased by almost the half compared to the control (*P* < 0.01). Differently, *ARF17* expression was similar to the control in the presence of 0.1 and 0.01 μM MeJA, whereas a significant (*P* < 0.01) upregulation of the gene (more than two-fold) was evident in the 10 μM MeJA-treated explants, independently of the genotype (Fig. 9). This increase in expression coupled with the formation of xylogenic cells in the place of ARs observed in these explants (Fig. 4e-f).

### The ethylene precursor ACC enhances AR-formation in the hypocotyls of the IBA + Kin-grown seedlings, but reduces it in the TCLs, however the ein3eil1 mutant is insensitive both in planta and in in vitro culture

**Fig. 10:**
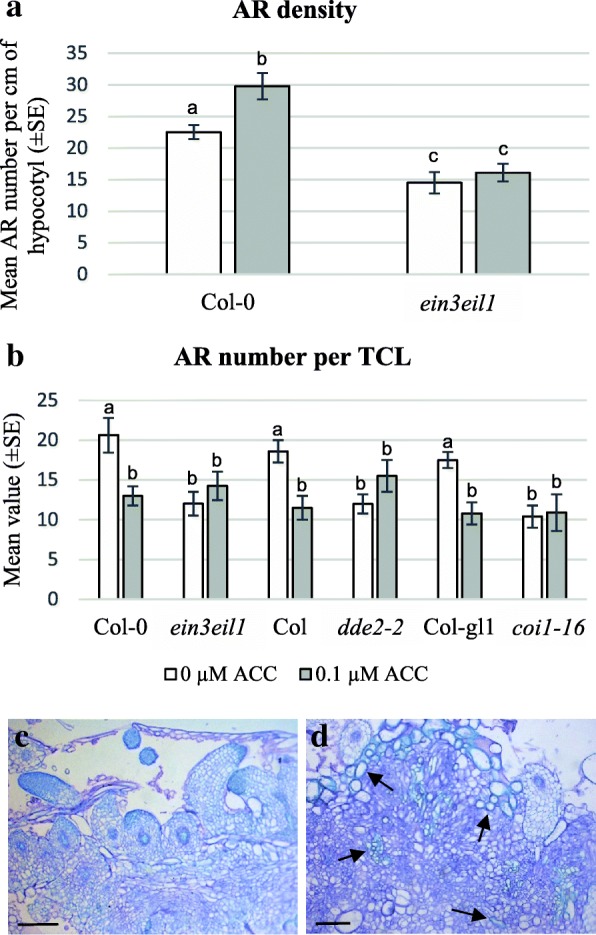
AR-formation in IBA + Kin-grown *ein3eil1* seedlings and TCLs, and TCLs of JA-mutants, with/without ACC-treatment. (**a**) Mean AR density, i.e. mean AR/ARP number per cm of hypocotyl (±SE), in Col-0 (WT) and *ein3eil1* seedlings grown under darkness in the presence/absence of 0.1 μM ACC, at 22 DAS. (**b**) Mean AR/ARP number per explant (±SE) in TCLs excised from *ein3eil1*, *dde2–2*, *coi1–16* mutant plants, and their WTs (Col-0, Col and Col-gl1, respectively), after 15 days of culture under darkness in the presence/absence of 0.1 μM ACC. (**c-d**) Histological images of Col-0 TCLs cultured in the absence of ACC (**c**) and with 0.1 μM ACC (**d**), at day 15. (**a**-**b**) Significant differences between treatments within the same genotype, or between each mutant and its WT under the same treatment, at least at *P <* 0.05 level*,* are indicated by different letters. The same letter within the same genotype, and between each mutant and its WT, shows no significant difference. Further statistical details are described in the text. *N* = 30 (**a**), *N* = 60 (**b**) (First replicate). (**c**) Section area of a TCL showing numerous ARs and a limited number of de novo formed xylary cells in the explant. (**d**) Detail of an ACC-cultured TCL showing a conspicuous differentiation of single, or grouped in clumps or strands, xylary cells (shown by arrows). Note that the lignified cell walls are in blue-green colour. Toluidine blue staining. (Images from the first replicate). Bars = 200 μm

Based on the known interaction between ET and JA in numerous responses [32], the ET-precursor ACC was applied to both seedlings and TCLs at the concentration of 0.1 μM, because it had been previously shown to enhance AR-formation in Arabidopsis seedlings grown under darkness in the presence of IBA alone [10]. As shown in Fig. 10a, the presence of ACC together with IBA and Kin significantly enhanced AR-formation in the hypocotyls, but this did not occur in the *ein3eil1* double mutant in accordance with its ET-insensitivity [10]. By contrast, the application of ACC resulted into a significant (*P* < 0.01) reduction in AR-formation in the WT TCLs, but again the double mutant was insensitive (Fig. 10b). The histological analysis revealed that while the AR-formation was reduced in the ACC-treated WT TCLs, the xylogenic response was enhanced in comparison with the explants cultured without ACC (Fig. 10c-d).

**Fig. 11:**
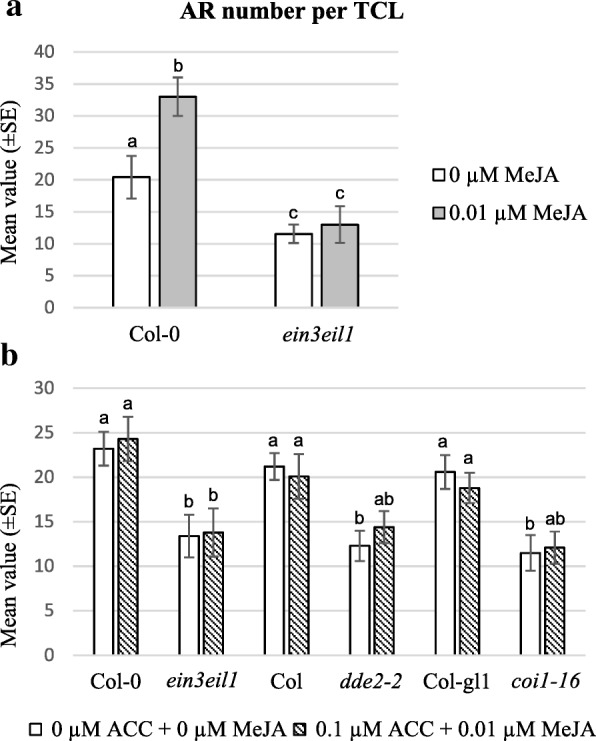
AR-formation in *ein3eil1* MeJA-treated TCLs, and *ein3eil1* and JA-mutant TCLs treated with ACC + MeJA. **(a)** Mean AR/ARP number per explant (±SE) in TCLs excised from Col-0 and *ein3eil1* plants, after 15 days of in vitro culture under darkness on IBA + Kin-medium, in the presence/absence of 0.01 μM MeJA. (**b**) Mean AR/ARP number per explant (±SE) in TCLs excised from *ein3eil1*, *dde2–2*, *coi1–16* mutant plants, and their WTs (Col-0, Col and Col-gl1, respectively), after 15 days of culture in vitro under darkness on IBA + Kin-medium, in the presence/absence of 0.1 μM ACC plus 0.01 μM MeJA. Significant differences between treatments within the same genotype, or between each mutant and its WT under the same treatment, at least at *P < 0.05* level*,* are indicated by different letters. The same letter within the same genotype, and between each mutant and its WT, shows no statistical difference. N = 60 (first replicate) (**a**,**b**)

The response of TCLs coming from the *dde2–2* and *coi1–16* mutants was also analyzed in the presence of ACC (0.1 μM) to investigate the interaction between JA (biosynthesis and perception) and ET-signalling in AR-formation. We decided to exclude *opr3* from the analysis, because the response of this mutant had suggested an effect of OPDA per se on AR-formation (Figs. 1b and 2). Differently from the WTs, the AR-response of *dde2–2* and of *coi1–16* TCLs was not significantly affected by ACC as for *ein3eil1* (Fig. 10b), suggesting an interaction of the endogenous JA and ET-signalling. To verify this possibility, *ein3eil1* TCLs were cultured under IBA + Kin with/without 0.01 μM MeJA, and the response compared with the WT (Col-0). The presence of MeJA enhanced AR-formation in Col-0 as in the other WT genotypes (Figs. 11a and 2, in comparison), but the AR-production of *ein3eil1* TCLs remained lower than that of its WT and not significantly affected by MeJA (Fig. 11a).

To better understand the interaction between JA and ET, the TCLs were cultured under IBA + Kin in the presence of both ACC (0.1 μM) and MeJA (0.01 μM). Interestingly, there was a compensative effect caused by the two hormones, because AR-production did not change significantly under the combined presence of ACC and MeJA in comparison with their absence in any genotype (Fig. 11b).

## Discussion

The results show that JA, at specific concentrations, positively affects either AR-formation or xylogenesis under darkness, acting in the same way in the intact hypocotyls and TCLs, grown under the same exogenous auxin plus cytokinin input.

### JA positively affects AR-formation in two different systems

**Fig. 12:**
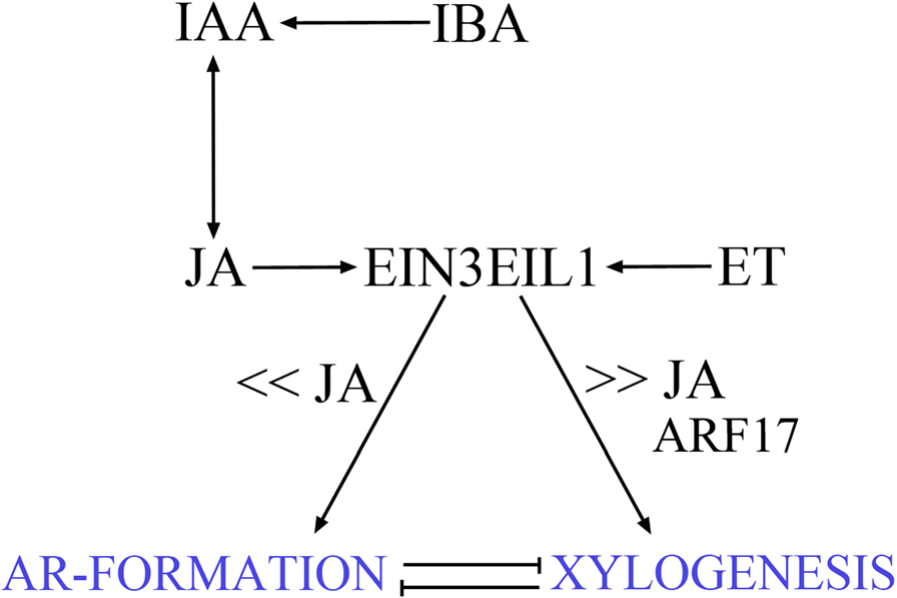
Model of the cross-talk among IAA, JA and ET in the realization of AR-formation and xylogenesis in IBA + Kin seedlings and TCLs of Arabidopsis grown under continuous darkness. Exogenous IBA induces IAA formation. IAA interacts with JA at early and late synthesis levels and at perception level. The capacity of the tissues to respond to JA also involves a cross-talk with ET-signalling through EIN3EIL1. The morphogenic response changes depending on JA levels. AR-formation needs low JA levels and does not require *ARF6* and *ARF8* changes in expression, whereas the competitive program of xylogenesis needs high JA-levels and the activity of *ARF17*. Further details on the text

It is known that different experimental systems can differently affect the rooting ability in Arabidopsis [5]. Also the two systems analyzed in the present research are considerably different. In fact, the hypocotyl is part of an entire seedling, in which, of course, the endogenous hormonal content is that of a whole plant. By contrast, TCLs are tissues excised from the inflorescence stem, i.e. a wounded system, which has been separated by the hormonal context of the whole plant, and in which wounding-related compounds, such as JAs [43] become soon active (present results). Under continuous darkness, in the intact Arabidopsis hypocotyls the endogenous auxin content is sufficient to induce an AR-response, even if limited ([8] and present results). This is not the case for the dark-grown TCLs, which are unable to form ARs under HF conditions [6], and totally devoid of any auxin at culture onset ([7], and present results). However, present results show that the JA deriving by the demethylation of MeJA, applied at 0.01 μM in combination with IBA + Kin, enhances AR-formation in both systems, and in all the WT genotypes. The common result in the two systems seems to exclude that the AR-response of the TCLs is induced by the JA formed in response to wounding. To verify this hypothesis, the endogenous levels of JA, JA-Ile, and OPDA were monitored in the TCLs. All the three compounds were detected in the WT TCLs at very high levels soon after excision, but their levels rapidly declined, remaining low during all the culture period leading to the first AR-cell cluster formation (days 1–5). However, in comparison with 0 MeJA, small, but significant, increases in JA occurred at each day in the presence of 0.01 μM MeJA, i.e. the treatment promoting AR-formation. Moreover, the absence of an AR-increase with a ten-fold higher concentration of MeJA (0.1 μM) in comparison with the treatment without MeJA, and the reduction/absence of AR-formation caused by the micromolar concentration (10 μM), clearly support that JA in combination with IBA + Kin is an enhancer of AR-formation, but at specific low levels (Fig. 12).

### JA is a co-mediator of IAA in AR-formation, with early synthesis and signalling involved

Our recent results have shown that, both *in planta* and in TCLs, the exogenous IBA must be converted into IAA to form ARs, and that IAA biosynthesis by the anthranilate synthase activity is required for AR-promotion in IBA-treated Arabidopsis dark-grown seedlings and TCLs [7, 10]. Present results show that the endogenous IAA levels increase in the TCLs at the time of the first AR-cell cluster formation (day 5), under 0.01 μM MeJA, and in parallel with JA increase. In accordance, a significant increase of free IAA levels has been observed treating Arabidopsis plants for 48 h with MeJA [44]. Taken together, present and past results suggest that JA is involved in AR-formation by positively affecting the endogenous IAA levels necessary for the process. The reduction in AR-response in the TCLs from the *dde2–2* and *coi1–16* mutants indicates that both an early step in biosynthesis and the perception of JA are involved in this co-mediation with auxin. In contrast with these data, a negative role of JA in de-etiolation-induced AR formation in intact hypocotyls of Arabidopsis has been reported, and put in relation with JA-signalling through COI1 [18]. The different result might be explained by the different hormonal and environmental conditions, i.e., absence of exogenous hormones vs IBA + Kin presence and light vs continuous darkness ([18] and present results in comparison), in accordance with the well known interaction of JA with other phytohormones, and the negative effects of JA on numerous light-induced genes [15, 45].

Interestingly, the response of the late-biosynthesis mutant *opr3* is different from that of the early-biosynthesis mutant *dde2–2*, because *opr3* shows an AR-response comparable with that of its WT, both *in planta* and in TCLs. The mutant *opr3* has been reported to be JA-deficient but OPDA-accumulating [15]. Present results suggest that OPDA may positively affect AR-formation in intact hypocotyls and TCLs through a different way from JA-signalling pathway. In accordance, OPDA is not an active ligand in COI1–JAZ pull-down assays [24], and OPDA-specific gene expression and JA-independent roles of OPDA have been reported [15, 46]. However, the small amount of JA/JA-Ile early detected in *opr3* mutant might also contribute to AR formation. In fact, an OPR3-independent pathway for JA/JA-Ile production has been reported for this mutant [47]. Even though it is not clear how much this pathway contributes to JA production in the WT, it might explain some of the results attributed basically to OPDA accumulation alone.

### MeJA enhances xylogenesis in TCLs at a concentration many-fold higher than that promoting AR-formation, and the expression of the AR-repressor gene ARF17 is involved

The ectopic/extra formation of xylem *in planta* is a type of xylogenic response [9, 11]. Interestingly, JA has been recently demonstrated to induce extra xylem in the roots of Arabidopsis Col-0 [19]. Moreover, *coi1–1* and *jasmonate resistant1–1* mutants, both involved in JA signalling, do not form extra xylem in response to JA, whereas *opr3* forms it [19]. In the same system, JA effect is dose-dependent, because about 15% of WT plants develop extra xylem when treated with 1 μM JA, whereas their percentage increases up to 60% with 10 μM JA [19]. Our results show that also the xylogenesis in Arabidopsis TCLs is under the control of JA (Fig. 12). However, as in the case of AR-formation, specific JA levels are necessary for triggering this program, and they seem to be higher than those necessary for rooting. In fact, as revealed by the present histological analysis, MeJA, at 10 μM, i.e. at a concentration 1000-fold higher than that enhancing AR-formation, is able to promote xylogenesis, in accordance with the promotion of extra xylem *in planta* by the same concentration [19]. However, the JA level stimulating xylogenesis might be different in the different species. In fact, the concentration of 10 μM MeJA is not xylogenesis-inductive in tobacco TCLs, in which 0.1 μM is able instead to induce the process [16].

It has been reported that cytokinin diminishes the effect of JA on xylem development in Arabidopsis seedlings [19]. This antagonistic effect does not seem to occur in Arabidopsis TCLs, because our preliminary results show that xylogenesis under 10 μM JA is similarly enhanced in both IBA + Kin and IBA alone cultured explants (Fig. 4f and Additional file 5: Figure S4 in comparison). A detailed quantitative study is in progress for a deep insight in the xylogenic response *in planta* and TCLs.

Among Arabidopsis ARF proteins that mediate auxin-induced gene activation [48], ARF6 and ARF8 induce JA biosynthesis in reproductive organs with partially overlapping functions [27]. Both genes are strongly expressed in Arabidopsis hypocotyls in light conditions, whereas *ARF17* expression is reduced [26]. Present histological observations and RT-qPCR-analyses show that under continuous darkness *ARF6* and *ARF8* expression remains localized in the ARP/AR tips independently of MeJA application, and *ARF17* expression is absent in the ARPs/ARs. However, interestingly, *ARF17::GUS* signal is present in the xylogenic zones. In accordance, even if the expression signal of *ARF17* is very low in the hypocotyls under light, it remains detectable in the vascular cells [26]. Our results show that the expression of *ARF17* increased in the TCLs in the presence of 10 μM MeJA, i.e. the concentration enhancing xylogenesis. This suggests a promoting role of ARF17 on xylogenesis in competition with AR-formation (Fig. 12), in accordance with its previously reported role of negative AR-regulator [26]. Interestingly, the TCLs of the *opr3* mutant showed the same expression pattern of the WT, with the same increase in *ARF17* expression under 10 μM MeJA. Coupled with the extra xylem formation observed in *opr3 in planta* with 10 μM JA [19], the OPDA, present in this JA deficient mutant, might induce per se not only AR-formation, but also xylogenesis. Of course, further research is necessary, because information about OPDA-activated networks is still very limited.

### JA–ET antagonistic interaction in AR-formation in TCLs involves EIN3/EIL1

It has been reported that JA and ET control plant defence in an interdependent manner, however, they also mutually antagonize certain of each others’ functions in morphogenesis, for example, in apical hook formation in etiolated seedlings [32]. In Arabidopsis, the enhanced AR-response *in planta* in the presence of the ET precursor ACC combined with IBA + Kin here observed is in accordance with previous data on AR-increase in etiolated seedlings caused by IBA alone in combination with ACC [10]. In addition, by the use of the *ein3eli1* mutant, impaired in ET perception, both previous [10] and present data show that ET-action involves the activity of the EIN3/EIL1 network. The same network has been demonstrated to be positively involved in xylogenesis *in planta* [11].

In TCLs treated with 0.01 μM MeJA, it is here shown that ACC, combined with IBA + Kin, reduces AR-formation by enhancing xylogenesis. This suggests an antagonism between JA and ET in the control of AR-formation in the TCLs, because xylogenesis competition. The low and unchanged AR-response of *ein3eil1*, under either 0.01 μM MeJA or 0.1 μM ACC, together with the compensation between the promoting AR-action by JA, and the reducing action by ET, observed in the combined treatment in the WT TCLs, suggests that EIN3 and EIL1 are the link between the action of the two hormones. In accordance, it has been reported that the molecular mechanisms for JA–ET antagonistic interactions are based on the mutual inhibitions between two branches of JA-activated transcription factors, one of which is EIN3/EIL1 [49, 50]. Thus, EIN3/EIL1 might represent the critical link between JA and ET in the control of AR-formation vs xylogenesis.

## Conclusions

In conclusion, results collectively uncover a critical function of the crosstalk between JA and ET-signalling in the auxin-induced AR-formation occurring under darkness in intact hypocotyls and in the in vitro cultured TCLs, involving a competitive modulation of xylogenesis (Fig. 12). Approaches similar to those here developed for Arabidopsis might be useful to improve knowledge about mechanisms in common between AR-formation *in planta* and in cuttings, and find a way to optimize conditions for better rooting of recalcitrant species by repressing the competitive realization of xylogenesis.

## Additional files

Additional file 1: **Figure S1.** Positive and negative controls of JA/JA-Ile immunolocalization. (**a**) Positive control: Ws TCL cultured under darkness on IBA + Kin medium for 15 days, fixed in 500 μM JA in 4% (*w*/*v*) EDC before immunolocalization procedure. (**b**) Negative control: Ws TCL cultured under darkness on IBA + Kin medium for 15 days, with sections not incubated with the anti-JA primary antibody during the immunolabeling procedure. (**a**-**b**) 5 μm thick sections observed under the epifluorescence microscope. Bars = 20 μm. (JPG 185 kb)

Additional file 2: **Table S1.** Primer sequences. List of sequences of the primers used for quantifying *ARF6*, *ARF8*, *ARF17* expression in Ws (WT) and *opr3* TCLs by RT-qPCR. (PDF 62 kb)

Additional file 3: **Figure S2.** Germination of different genotypes (HF-, IBA + Kin-media) and AR density in WT seedlings (HF-medium). (**a-b**) Percentage of germination of *dde2–2*, *opr3* and *coi1–16* mutant seeds, and of their WTs (Col, Ws and Col-gl1, respectively), on a medium without IBA and Kin (HF medium, **a**) or containing 10 μM IBA and 0.1 μM Kin (IBA + Kin medium, **b**), either in the absence of MeJA (0 μM MeJA) or in the presence of 0.01 μM or 0.1 μM MeJA. (**c**) Mean AR density, i.e. mean ARP/AR number per cm of hypocotyl (±SE), of seedlings of three WT genotypes grown under darkness on HF medium, at 22 DAS. ^a,^ *P* < 0.05 difference with respect to the other genotypes. No letter indicates no significant difference. *N* = 30 (**a**, **b**, **c**). (PDF 87 kb)

Additional file 4: **Figure S3.** Expression patterns of *ARF6::GUS*, *ARF8::GUS*, *ARF17::GUS* in dark-grown seedlings grown with/without 0.01 μM MeJA, at 22 DAS. (**a-b**) GUS signal detected in the AR apex of *ARF6::GUS* from two different replicates. (**c-d**) *ARF8::GUS* expression in AR apices of seedlings from different replicates. (**e**-**f**) Absence of GUS signal in *ARF17::GUS* AR apices from seedlings of different replicates. (**g**) Expression signal in the vasculature (arrow) near the ARP, in the hypocotyl of an *ARF17::GUS* seedling. (**h**-**i**) *ARF17::GUS* expression in the vascular connection between ARs and their LRs (arrow) in seedlings from different replicates. Whole-mount seedlings observed under light microscopy. Bars = 100 μm (**a**-**c**), 50 μm (**d**-**i**). (JPG 1392 kb)

Additional file 5: **Figure S4.** Xylogenesis obtained in dark-grown TCLs cultured with IBA alone (10 μM) combined with MeJA (10 μM). Detail of a longitudinal radial section of a Ws TCL showing xylary elements (arrows) differentiating from the endodermis-derived cells (day 15). Bar = 50 μm. (JPG 593 kb)

## Abbreviations

ACC: 1-aminocyclopropane-1-carboxylic acid
AR: Adventitious root
ARP: Adventitious root primordium
DAS: Days after stratification
ET: Ethylene
FW: Fresh weight
IAA: Indole-3-acetic acid
IBA: Indole-3-butyric acid
JA: Jasmonic acid
Kin: Kinetin
LM: Light microscopy
LR: Lateral root
MeJA: Methyl jasmonate
OPDA: 12-oxophytodienoic acid
PR: Primary root
TCL: Thin cell layer
TF: Transcription factor
WT: Wild type
