# Supplementary figures and images for: Jasmonate promotes auxin-induced adventitious rooting in dark-grown Arabidopsis thaliana seedlings and stem thin cell layers by a cross-talk with ethylene signalling and a modulation of xylogenesis
Source: BMC Plant Biol. 2018 Sep 6;18:182. doi: 10.1186/s12870-018-1392-4 (PMC6127917; doi:10.1186/s12870-018-1392-4)

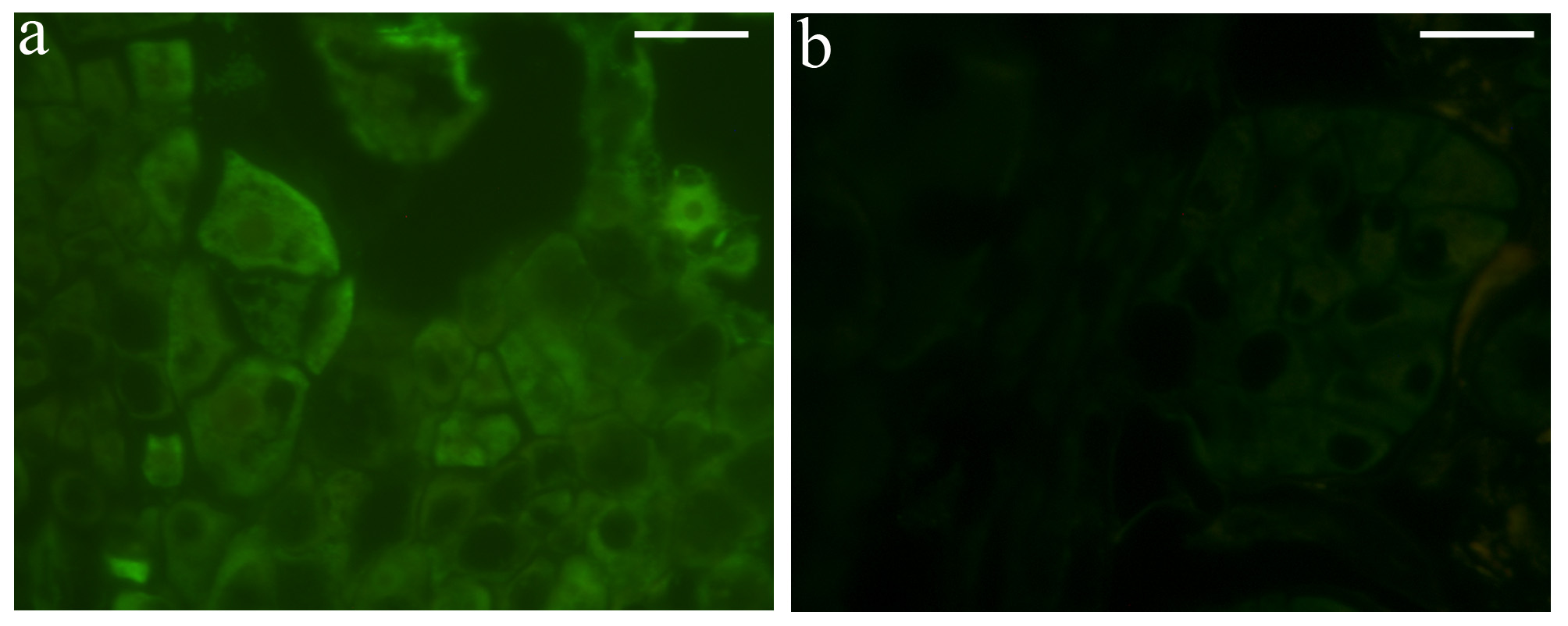

Supplement: Supplementary file 1 — Figure S1. Positive and negative controls of JA/JA-Ile immunolocalization. (a) Positive control: Ws TCL cultured under darkness on IBA + Kin medium for 15 days, fixed in 500 μM JA in 4% (w/v) EDC before immunolocalization procedure. (b) Negative control: Ws TCL cultured under darkness on IBA + Kin medium for 15 days, with sections not incubated with the anti-JA primary antibody during the immunolabeling procedure. (a-b) 5 μm thick sections observed under the epifluorescence microscope. Bars = 20 μm. (JPG 185 kb) [file 12870_2018_1392_MOESM1_ESM.jpg]

Figure S2

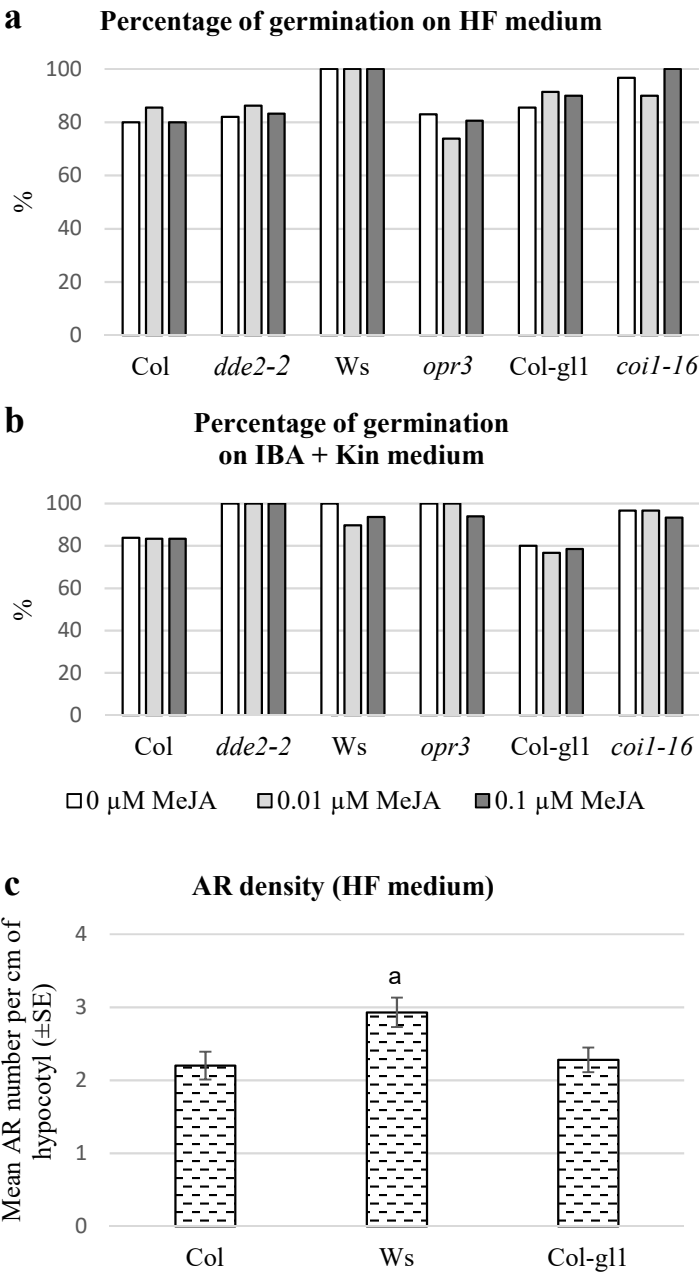

Supplement: Supplementary file 3 — Figure S2. Germination of different genotypes (HF-, IBA + Kin-media) and AR density in WT seedlings (HF-medium). (a-b) Percentage of germination of dde2–2, opr3 and coi1–16 mutant seeds, and of their WTs (Col, Ws and Col-gl1, respectively), on a medium without IBA and Kin (HF medium, a) or containing 10 μM IBA and 0.1 μM Kin (IBA + Kin medium, b), either in the absence of MeJA (0 μM MeJA) or in the presence of 0.01 μM or 0.1 μM MeJA. (c) Mean AR density, i.e. mean ARP/AR number per cm of hypocotyl (±SE), of seedlings of three WT genotypes grown under darkness on HF medium, at 22 DAS. a, P < 0.05 difference with respect to the other genotypes. No letter indicates no significant difference. N = 30 (a, b, c). (PDF 87 kb) [file 12870_2018_1392_MOESM3_ESM.pdf]

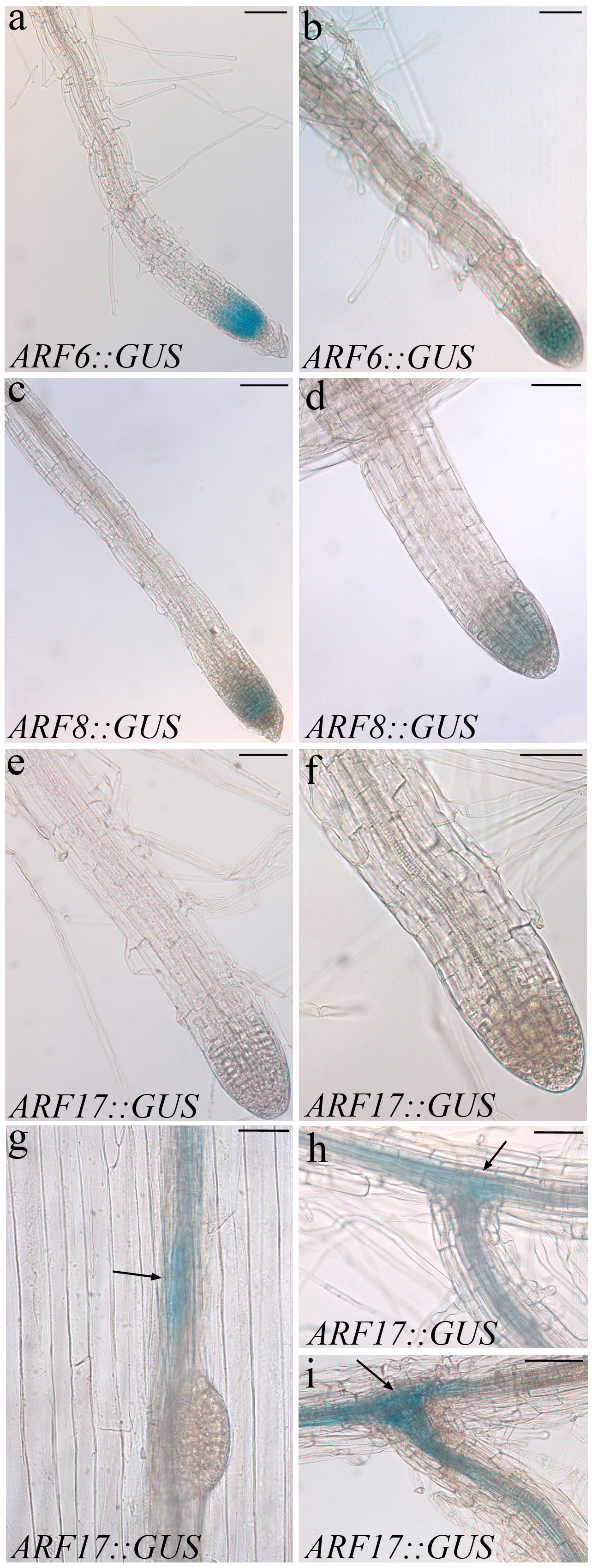

Supplement: Supplementary file 4 — Figure S3. Expression patterns of ARF6::GUS, ARF8::GUS, ARF17::GUS in dark-grown seedlings grown with/without 0.01 μM MeJA, at 22 DAS. (a-b) GUS signal detected in the AR apex of ARF6::GUS from two different replicates. (c-d) ARF8::GUS expression in AR apices of seedlings from different replicates. (e-f) Absence of GUS signal in ARF17::GUS AR apices from seedlings of different replicates. (g) Expression signal in the vasculature (arrow) near the ARP, in the hypocotyl of an ARF17::GUS seedling. (h-i) ARF17::GUS expression in the vascular connection between ARs and their LRs (arrow) in seedlings from different replicates. Whole-mount seedlings observed under light microscopy. Bars = 100 μm (a-c), 50 μm (d-i). (JPG 1392 kb) [file 12870_2018_1392_MOESM4_ESM.jpg]

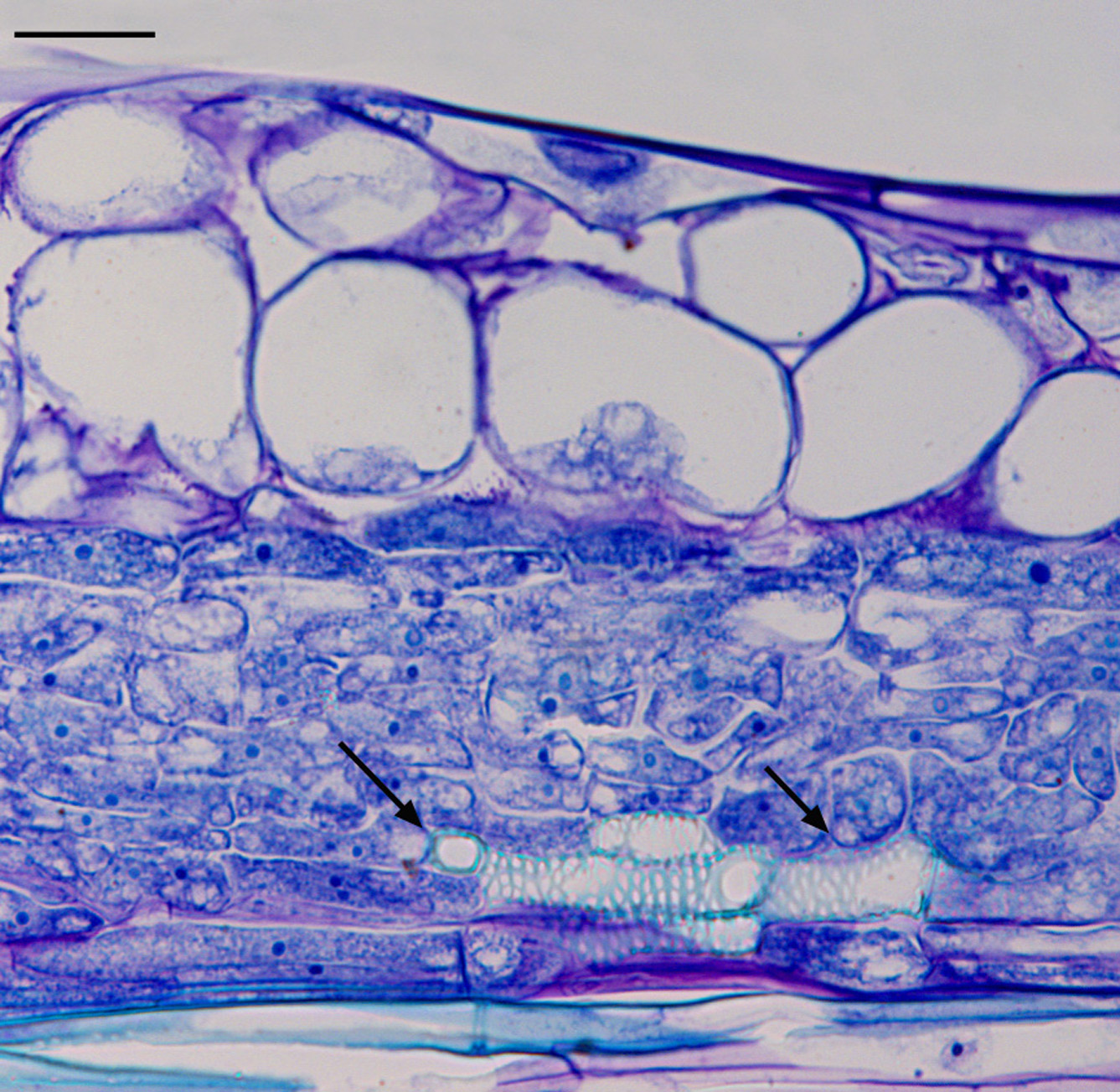

Supplement: Supplementary file 5 — Figure S4. Xylogenesis obtained in dark-grown TCLs cultured with IBA alone (10 μM) combined with MeJA (10 μM). Detail of a longitudinal radial section of a Ws TCL showing xylary elements (arrows) differentiating from the endodermis-derived cells (day 15). Bar = 50 μm. (JPG 593 kb) [file 12870_2018_1392_MOESM5_ESM.jpg]
