# Supplementary material for: Jasmonate promotes auxin-induced adventitious rooting in dark-grown Arabidopsis thaliana seedlings and stem thin cell layers by a cross-talk with ethylene signalling and a modulation of xylogenesis
Source: BMC Plant Biol. 2018 Sep 6;18:182. doi: 10.1186/s12870-018-1392-4 (PMC6127917; doi:10.1186/s12870-018-1392-4)
Supplement: Supplementary file 2 — Table S1. Primer sequences. List of sequences of the primers used for quantifying ARF6, ARF8, ARF17 expression in Ws (WT) and opr3 TCLs by RT-qPCR. (PDF 62 kb) [file 12870_2018_1392_MOESM2_ESM.pdf]

Table S1

List of sequences of primers used for quantifying target genes by RT-qPCR.

Gene name	Forward primer	Reverse primer
ARF6/At1g30330	CAAAGTTTAGCAGCTACCACGA	ACGTCGTTCTCTCGGTCAAC
ARF8/At5g37020	TTTGCTATCGAAGGGTTGTTG	CATGGGTCATCACCAAGGA
ARF17/At1g77850	GCACCTGATCCAAGTCCTTC	GGTGAATAGCTGGGGAGGAT
